# NAP1-RELATED PROTEIN1 and 2 negatively regulate H2A.Z abundance in chromatin in Arabidopsis

**DOI:** 10.1038/s41467-020-16691-x

**Published:** 2020-06-08

**Authors:** Yafei Wang, Zhenhui Zhong, Yaxin Zhang, Linhao Xu, Suhua Feng, Shima Rayatpisheh, James A. Wohlschlegel, Zonghua Wang, Steven E. Jacobsen, Israel Ausin

**Affiliations:** 1 State Key Laboratory of Crop Stress Biology for Arid Areas and College of Life Sciences, Northwest A&F University, Yangling, 712100 Shaanxi China; 2 Department of Molecular, Cell and Developmental Biology, University of California, Los Angeles, CA 90095 USA; 3 State Key Laboratory of Ecological Pest Control for Fujian and Taiwan Crops, College of Plant Protection, Fujian Agriculture and Forestry University, Fuzhou, 350002 China; 4 Haixia Institute of Science and Technology, Fujian Agriculture and Forestry University, Fuzhou, 350002 China; 5 Leibniz Institute of Plant Genetics and Crop Plant Research (IPK), OT Gatersleben, Stadt Seeland, 06466 Germany; 6 Department of Biological Chemistry, David Geffen School of Medicine, University of California, Los Angeles, CA 90095 USA; 7 Howard Hughes Medical Institute, University of California, Los Angeles, CA 90095 USA

**Keywords:** Histone variants, Nucleosomes, Plant genetics

## Abstract

In eukaryotes, DNA wraps around histones to form nucleosomes, which are compacted into chromatin. DNA-templated processes, including transcription, require chromatin disassembly and reassembly mediated by histone chaperones. Additionally, distinct histone variants can replace core histones to regulate chromatin structure and function. Although replacement of H2A with the evolutionarily conserved H2A.Z via the SWR1 histone chaperone complex has been extensively studied, in plants little is known about how a reduction of H2A.Z levels can be achieved. Here, we show that NRP proteins cause a decrease of H2A.Z-containing nucleosomes in Arabidopsis under standard growing conditions. *nrp1-1 nrp2-2* double mutants show an over-accumulation of H2A.Z genome-wide, especially at heterochromatic regions normally H2A.Z-depleted in wild-type plants. Our work suggests that NRP proteins regulate gene expression by counteracting SWR1, thereby preventing excessive accumulation of H2A.Z.

## Abstract (web summary)

The histone variant H2A.Z is deposited by the SWR1 complex to replace H2A in Arabidopsis, but the mechanism of H2A.Z removal is unclear. Here, the authors show that NRP proteins can regulate gene expression by counteracting SWR1 and prevent excessive accumulation of H2A.Z.

## Introduction

DNA encodes the genetic information in the cell, but in all eukaryotes, DNA is rarely found naked but usually packaged into a higher-order structure termed chromatin, which is composed of DNA and histones. The basic unit of the chromatin is the nucleosome, consisting of 147 base pairs of DNA wrapped around the octamer, which is formed by one Histone3—Histone4 tetramer (H3-H4)x2 and two Histone2 dimers (H2A-H2B)x2^1^.

Canonical histones can be replaced by histone variants in a replication-independent manner. Among H2A variants, H2A.Z is one of the most evolutionary conserved^2^, and has important implications not only in the regulation of gene expression but also in genome stability, cell cycle, DNA repair, recombination, female meiosis, response to high temperatures as well as different kinds of biotic and abiotic stresses^3–11^. H2A.Z is typically found in the arms of the chromosomes and mainly excluded from chromocenters^12^. In particular, H2A.Z is usually enriched at the first nucleosome after the transcriptional start site (+1 nucleosome), although it can be found at a lower level in gene bodies^13^. Regarding gene regulation, it has been proposed that the presence of H2A.Z at the +1 nucleosome is associated with transcriptional activity, whereas H2A.Z has a repressive effect on transcription when located across gene bodies^8^. In addition, a recent report proposes that the effect of H2A.Z on transcription depends on the post-translational modification it may bear, where monoubiquitylation of H2A.Z would have a repressive effect on transcriptional activity while acetylated H2A.Z would serve as an activating mark^14^.

H2A.Z is deposited by the Swi/Snf2 Related1 (SWR1) complex, which is a chromatin remodeler that specifically replaces the H2A-H2B dimer with the H2A.Z-H2B dimer in a stepwise reaction that requires ATP^15,16^. It is still uncertain what is the precise mechanism by which SWR1 is recruited to the chromatin. However, in yeast, acetylation of H4 located at promoters is required for the proper deposition of H2A.Z at the +1 nucleosome^17–19^. Related to that, two recent reports suggest that METHYL-CpG-BINDING DOMAIN9 may guide SWR1 to open chromatin regions nearby active genes in Arabidopsis^20,21^.

Nucleosome assembly protein 1 (NAP1) was first described in *Xenopus leavis* as an H2A/H2B histone chaperone that promotes nucleosome assembly in vitro^22^. Subsequently, NAP1 was shown to be involved in H2A/H2B trafficking and to facilitate nucleosome disassembly^23,24^. NAP1 is evolutionarily conserved from yeast to humans. In Arabidopsis, the NAP1 family consists of six members with similarity to the yeast H2A/H2B histone chaperone NAP1 and human SET/TAF-Iβ^25^: NAP1;1, NAP1;2, NAP1;3, NAP1;4, as well as the two closely related orthologues NAP1-RELATED PROTEIN 1 (NRP1) and NRP2. Interestingly, NRP1 and 2 are the two proteins that have diverged the most from the founding member AtNAP1^26^, which raises the possibility of some degree of functional diversity. In Arabidopsis, NRP proteins have been implicated in several biological processes, including cell-cycle control, root meristem formation, heat tolerance, DNA repair, somatic homologous recombination, and genome defense under genotoxic stress^25,27–29^. NRP proteins are localized mainly in the nucleus and bind H2A, H2B, H3, and H4 histones^25,30^. However, a molecular mechanism for these proteins has not been clearly established.

Here, we show that NRP proteins genetically interact with the core components of SWR1 and associate with H2A.Z in vivo. We have also found that in *nrp1-1 nrp2-2*, H2A.Z shows an altered pattern and excessive levels compared to wild-type, a phenotype that is consistent with NRP1 localization. Together these data suggest that NRP proteins counteract the activity of the SWR1 complex and contribute to the dynamic regulation of H2A.Z in Arabidopsis.

## Results

### NRPs regulate the expression of developmental key genes

**Fig. 1:**
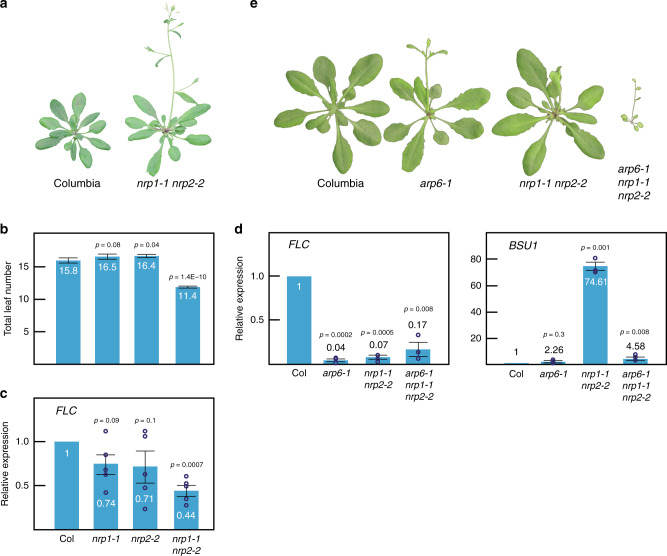
The phenotype of *nrp1-1 nrp2-2* double mutants. **a** Columbia and *nrp1-1 nrp2-2* plants grown 5 weeks under long-day conditions. **b** Flowering time of Columbia, *nrp1-1, nrp2-2*, and *nrp1-1 nrp2-2* plants expressed as the total number of leaves under long-day conditions. Average from 12 (*nrp1-1* and *nrp2-2*) or 20 (Columbia and *nrp1-1 nrp2-2*) plants ± standard error (SE) is shown. **c** Relative expression of *FLC* in Columbia, *nrp1-1, nrp2-2*, and *nrp1-1 nrp2-2* backgrounds measured by RT-PCR. Error bars represent standard error. This experiment was repeated under the same conditions yielding similar results. **d** Relative expression of *FLC* and *BSU1* in Columbia, *arp6-1*, *nrp1-1 nrp2-2*, and *arp6-1 nrp1-1 nrp2-2* backgrounds measured by RT-PCR. Error bars represents standard deviation. *UBQ10* was used as an internal control. **e** Morphological phenotype of 5 weeks old Columbia, *arp6-1*, *nrp1-1 nrp2-2*, and *arp6-1 nrp1-1 nrp2-*2 plants grown under long-day conditions. **b**–**d** Two-tailed, paired Student’s *t* test was used to determine *p*-value. Source data underlying Fig. 1b–d are provided as a Source Data file.

The *nrp1-1 nrp2-1* double mutant shows a root developmental defect as the only reported apparent morphological phenotype^28^. The *nrp2-1* mutant carries a T-DNA insertion in a non-coding region^28^, but in this study, we have used *nrp2-2* allele instead, which carries a T-DNA insertion in the coding region and therefore it is likely a null allele. We found that *nrp1-1* and *nrp2-2* single mutants did not display any obvious morphological phenotype. However, the *nrp1-1 nrp2-2* double mutant showed a slightly early flowering phenotype that correlated with lower levels of *FLOWERING LOCUS C* (*FLC*) expression compared to wild-type (Fig. 1a–c). To find additional genes whose expression was affected by mutations in *NRP* genes, we performed RNA-Seq in Columbia, *nrp1-1*, *nrp2-2*, and the *nrp1-1 nrp2-2* double mutant. Among the misregulated genes, we found that *BRI1 SUPPRESSOR 1* (*BSU1*) transcript levels were dramatically increased in *nrp1-1 nrp2-2* double mutants relative to wild-type plants, which was in contrast with previous transcriptomic analyses using *nrp1-1 nrp2-1*^28^ (Fig. 1d). The phosphatase BSU1 dephosphorylates the kinase BRASSINOSTEROID INSENSITIVE2 (BIN2), which positively regulates the brassinosteroid signaling pathway^31^. Indeed, simultaneous disruption of *NRP1* and *2* suppressed phenotypes arising from *BIN2* overexpression, likely due to BSU1-mediated dephosphorylation of BIN2, since BIN2 protein levels were unaltered (Supplementary Fig. 1a, b). The kinase BRASSINOSTEROIDS INSENSITIVE1 (=BRI1) activates BSU1^32^. The weak mutant allele *bri1-119*, displays a pleiotropic phenotype including infertility, small dark green rosettes, inward curled leaves, and extra axillary inflorescences^33^, which was alleviated in the *nrp1-1 nrp2-2* background (Supplementary Fig. 1a), further supporting the overexpression of *BSU1* upon loss of NRP proteins.

### The SWR1 complex is required for *nrp1-1 nrp2-2* phenotype

To determine how NRP proteins regulate the expression of these genes, we examined genetic interactions with other histone chaperones. Specifically, we crossed the *nrp1-1 nrp2-2* double mutant with mutants encoding putative H2A-H2B chaperones. We found that *nrp1-1 nrp2-2* double mutants enhanced *arp6-1* phenotype when we looked at the overall morphological phenotype (Fig. 1e). Unexpectedly, transcript levels of *BSU1* were restored to nearly wild-type levels in the *arp6-1 nrp1-1 nrp2-2* triple mutant compared to the *nrp1-1 nrp2-2* double mutant. We did not observe a full restoration of the expression of *FLC*, but only a slight recovery (Fig. 1d). These data suggest that ACTIN RELATED PROTEIN6 (ARP6) is required for the altered expression of these genes in the *nrp1-1 nrp2-2* mutant background.

ARP6 is a core component protein of the Arabidopsis SWR1 complex, which replaces H2A-H2B by H2A.Z-H2B in an ATP-dependent manner^16^. We hypothesized that SWR1 activity could be essential to explain the observed phenotypes. Indeed, mutants affecting other known components of the SWR1 complex, SERRATED LEAVES AND EARLY FLOWERING (SEF) and PHOTOPERIOD-INDEPENDENT EARLY FLOWERING1 (PIE1), when crossed to the *nrp1-1 nrp2-2* double mutant, yielded similar results (Supplementary Fig. 2). Also, combining the *nrp1-1 nrp2-2* double mutant with mutations in *HTA9* and *HTA11*, two of the three genes encoding H2A.Z in Arabidopsis, suppressed the up-regulation of *BSU1* in the *nrp1-1 nrp2-2* double mutant (Supplementary Fig. 2). Thus, H2A.Z is required for the increased expression of *BSU1* in the *nrp1-1 nrp2-2* background. We observed a much more severe phenotype when mutations in *NRP1* and *2* were combined with SWR1 core components compared to mutations in H2A.Z coding genes themselves (Supplementary Fig. 2). This might be due to the involvement of SWR1 complex components in cellular mechanisms other than H2A.Z deposition or the presence of wild-type HTA8 in the *hta9-1 hta11-1* double mutant.

### NRP proteins interact with H2A.Z in vivo

**Fig. 2:**
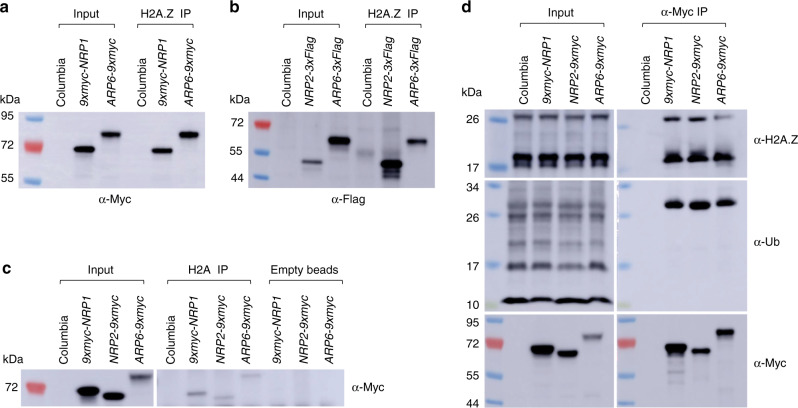
NRP proteins physically interact with H2A and H2A.Z in vivo. **a** and **b** α-H2A.Z co-immunopurification assays; α-H2A.Z immunoprecipitation lanes (=H2A.Z IP) show co-purification of H2A.Z with NRP1 (**a**) and NRP2 (**b**). **c** α-H2A co-immunopurification assays; α-H2A immunoprecipitation lanes (=H2A IP), show co-purification of H2A with NRP1 and NRP2. We show empty beads as a negative control. **d** NRPs co-immunopurification assays; α-Myc immunoprecipitation lanes (=Myc IP) show co-purification of NRP1 and NRP2 with both unmodified and monoubiquitinated H2A.Z. For each Western blot, the antibody used for detection is indicated either at the bottom, in the case of **a** and **b**, or to the right, in the case of **c** and **d**. In all cases, protein extracts from the same plants are included to confirm the identity of the co-precipitating band (=Input). We included ARP6 as a positive control. All co-immunopurification assays were repeated with similar results. Source data are provided as a Source Data file.

To further illuminate the molecular function of NRP proteins, we constructed Myc- and Flag-tagged versions of *NRP1* and *2* driven by their native 5′ regulatory regions for complementation analysis. We found that the *BSU1* overexpression phenotype of the *nrp1-1 nrp2-2* double mutant was complemented by the expression of N-terminally tagged 9xMyc-NRP1, as well as C-terminally, tagged NRP2-9xMyc, and NRP2-3xFlag (Supplementary Fig. 3). We subsequently performed experiments with these complementing constructs in their respective single mutant background. We used both *NRP2-3xFlag* and *NRP2-9xMyc* complementing lines for immunoprecipitation, followed by Mass Spectrometry. We found that NRP1 was present in all replicates in a nearly 1:1 ratio with NRP2, which is consistent with these two proteins forming heteromers in vivo^34^ (Table 1). We confirmed this interaction by co-immunoprecipitation assays in F_1_ lines expressing both NRP2-3xFlag and 9xMyc-NRP1 **(**Supplementary Fig. 4**)**. Given that NRP proteins share some degree of similarity with NAP1 and genetically interact with core components of Arabidopsis SWR1, we next tested whether they interact with either H2A or H2A.Z in vivo. Previous works showed that NRP1 could bind with H2A, H2B and H3 in pull-down assays^28^ as well as H2B, H3, H4, H2A-H2B, and H3-H4 tetramers as measured by ITC^30^. Consistently, co-IP assays confirmed that NRP1 and 2 physically interact with canonical H2A. In Addition, we found a strong interaction with the replacement variant H2A.Z in vivo (Fig. 2a–c). A recent study^14^ showed that H2A.Z can be monoubiquitinated in Arabidopsis; co-IP assays showed that both NRP proteins bind unmodified as well as monoubiquitinated H2A.Z in vivo (Fig. 2d).

**Table 1:** Mass spectrometric analyses of NRP2-3xFlag and NRP2-9xMyc affinity purifications.

Locus	Description	NRP2-Flag	NRP2-Myc	NRP2-Myc	NRP2-Myc	Col1	Col2	Col3	Col4
At1g18800	NRP2	530	217	270	433	0	0	0	0
At1g74560	NRP1	409	157	212	417	0	0	0	0
At4g31620	Transcriptional Factor B3 family protein	28	6	6	6	0	0	0	0

Total number of identified peptide spectra matched for each protein. Source data are provided as a Source Data file.

### *nrp1-1 nrp2-2* shows overaccumulation of H2A.Z

**Fig. 3:**
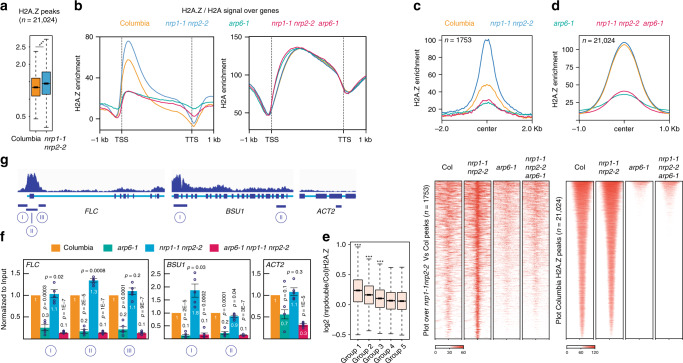
*nrp1-1 nrp2-2* double mutants display overaccumulation of H2A.Z. **a** Boxplot of log2 ratio of H2AZ/Input in Columbia and *nrp1-1 nrp2-2* over H2A.Z peaks defined in Columbia. *** indicates *p*-value of <2.2e^−16^ (Two-tailed paired Student’s *t* test). **b** Metaplot of H2A.Z/Input and H2A/Input in Columbia, *nrp1-1 nrp2-2*, *arp6-1*, and *arp6-1 nrp1-1 nrp2-2* over the gene body. **c** Plot and heatmap of Columbia, *nrp1-1 nrp2-2*, *arp6-1*, and *nrp1-1 nrp2-2 arp6-1* over *nrp1-1 nrp2-2* dependent H2A.Z peaks (*n* = 1753). **d** Plot and heatmap of H2A.Z peaks (*n* = 21,024) in Columbia, *nrp1-1 nrp2-2*, *arp6-1*, and *nrp1-1 nrp2-2 arp6-1*. **e** Boxplot showing the divergence of H2A.Z occupancy between Columbia and *nrp1-1 nrp2-2* at different groups of genes divided according to increasing expression levels. Center lines indicate the median, boxes show the 25th (bottom) and 75th (top) percentiles; whiskers extend to the minima and maxima. *** Indicates a *p*-value of <2.2e^−16^, <2.2e^−16^, and 9.57e^−6^ for Group 1, 2, and 3 respectively determined by two-tailed, paired Student’s *t* test. **f** The relative abundance of H2A.Z in Columbia, *arp6-1*, *nrp1-1 nrp2-2*, and *arp6-1 nrp1-1 nrp2-2* measured by ChIP-PCR at *FLC, BSU1*, and *ACT2*. Error bars represent standard error from five biological replicates. Two-tailed, paired Student’s *t* test was used to determine *p*-value. **g** The upper part of the *p*anel shows the distribution of H2A.Z ChIP reads across the genes immediately below. Schematic representation of the genes *FLC, BSU1*, and *ACT2*. Introns and exons are shown in light blue and navy blue, respectively. Roman numerals in **f** and **g** indicate the amplicon that was used by ChIP-qPCR analyses. Source data underlying Fig. 3a, e, f are provided as a Source Data file.

Given our findings that disruption of genes encoding H2A.Z or the SWR1 complex suppresses the phenotype of *nrp1-1 nrp2-2* at *BSU1*, and that NRP proteins can bind not only H2A but also H2A.Z, we hypothesized that NRP proteins might act to reduce H2A.Z locally. Western blot analysis suggested that global levels of H2A and H2A.Z were comparable in *nrp1-1 nrp2-2* and wild-type plants (Supplementary Fig. 5). To investigate changes in the levels of H2A.Z in *nrp1-1 nrp2-2* genome-wide, we performed H2A.Z Chromatin Immunoprecipitation followed by whole-genome sequencing (ChIP-Seq). First, we examined H2A.Z peaks defined in wild-type (*n* = 21,024) and observed significantly higher average occupancies of H2A.Z in the *nrp1-1 nrp2-2* double mutant compared to wild-type (Fig. 3a). Moreover, metagene analysis also showed a general accumulation of H2A.Z in the double mutant, except in regions where H2A.Z occupancy is naturally low, such as within and immediately upstream of the transcription start sites and transcription termination sites (TSS and TTS) (Fig. 3b). Additionally, we found 1753 highly significant H2A.Z peaks that were dependent on *nrp1-1 nrp2-2* (*p*-value < 2.2e-16) (Fig. 3c). These peaks fall mainly in regions annotated as coding genes (1467 coding / 286 TEs Vs 1334 coding / 419 TEs that would be expected from a randomly shuffled control). Also, consistent with our genetic data, H2A.Z occupancy in the *arp6-1 nrp1-1 nrp2-2* triple mutant was slightly higher than that in the *arp6-1* single mutant (Fig. 3c and d). We validated H2A.Z ChIP-Seq bioinformatic analyses by ChIP-qPCR at selected loci (Supplementary Fig. 6).

The increased occupancy of H2A.Z observed in *nrp1-1 nrp2-2* occurred predominantly at lowly expressed genes (Fig. 3e), which is consistent with the fact that lowly expressed genes typically show high levels of H2A.Z^14,35^.

Next, we performed ChIP-qPCR to assess the occupancy of H2A.Z at two putative NRP1/2 targets; *FLC* and *BSU1* (Fig. 3f). The analysis showed that compared to wild-type, *nrp1-1 nrp2-2* double mutant displayed higher occupancy of H2A.Z at both loci, within regions that were previously defined as H2A.Z peaks^13^ (Fig. 3g and Supplementary Fig. 7). However, we did not observe any significant enrichment in low abundance H2A.Z regions nor the negative control *ACTIN2* (Fig. 3g). These data suggest that NRP proteins might mitigate excessive deposition of H2A.Z.

### SWR1 and NRPs have opposite effects on transcription

Since it has been established that low levels of H2A.Z at the first intron of *FLC* are correlated with a reduction in its expression^12,36^, it was striking that we detected higher H2A.Z levels in the first intron of *FLC* in *nrp1-1 nrp2-2* compared to wild-type and observed a reduced expression of *FLC* (Fig. 3f, g, and Supplementary Fig. 7). We searched for a possible explanation and found that in *nrp1-1 nrp2-2* double mutant chromatin in the first intron of *FLC* appears to be denser compared to wild-type. Although we do not know exactly the precise mechanism, it is possible that compact chromatin around *FLC* regulatory region might have a negative impact on its transcription. We have also tested the chromatin density of *BSU1* in *nrp1-1 nrp2-2* and found no significant difference compared to wild-type (Supplementary Fig. 8).

To better understand the effects of NRP proteins on transcription, we first focused on the H2A.Z peaks that were dependent on *nrp1-1 nrp2-2*. This analysis showed that the subset of genes annotated as coding with increased H2A.Z occupancy immediately after the TSS (+1 nucleosome), but not other regions, displayed a slight but significant up-regulation in *nrp1-1 nrp2-2* (Supplementary Fig. 9a). This agrees with the fact that lowly expressed genes with high H2A.Z in both the gene body and TSS, on average, show higher expression than lowly expressed genes with high H2A.Z levels at the gene body but lower H2A.Z levels at the TSS^14,35^. Next, we examined the global effect of *nrp1-1 nrp2-2* on transcription. We divided all genes according to expression levels and noted that, generally, only highly expressed genes were downregulated by *nrp1-1 nrp2-2* (Supplementary Fig. 9b). This is consistent with previous studies reporting that highly expressed genes have very low H2.A.Z levels while moderately expressed genes have higher H2A.Z levels^14^. We then compared the transcriptomes of *arp6-1* and *nrp1-1 nrp2-2*. Consistent with the phenotype, the overall effect on transcription of *arp6-1* was larger than that of *nrp1-1 nrp2-2*. When comparing both transcriptomes, we observed that the number of genes that were downregulated in one but up-regulated in the other (580/317) were marginally higher than the number of genes that were upregulated or downregulated in both (519/272) (Supplementary Fig. 9c). These data suggest that ARP6 and NRP proteins may perform a counteracting activity in the cell.

### *nrp1-1 nrp2-2* does not affect global distribution of H2A

Since NAP1 was originally described as an H2A-H2B chaperone and NRP proteins have been shown to bind H2A^28^ (Fig. 2a–c), we wanted to explore if NRP proteins could have any effect on the deposition of canonical H2A. We previously showed that global levels of H2A were not significantly different between wild-type and *nrp1-1 nrp2-2* double mutant (Supplementary Fig. 5). To get more detailed information about H2A abundance and genome-wide distribution, we performed two independent biological replicates of H2A ChIP-Seq. The analysis of metagene plots revealed that the overall distribution of H2A in Columbia was nearly identical to that of *nrp1-1 nrp2-2* (Fig. 3b), suggesting that NRP proteins may not play an essential role in the dynamics of canonical H2A. We did notice an increase of H2A levels in *arp6-1* and *arp6-1 nrp1-1 nrp2-2* towards the 5′ end (Fig. 3b). This increase is likely because H2A-H2B replacement by H2A.Z-H2B in *arp6-1* backgrounds is severely compromised, meaning that an H2A increase could be expected at regions where H2A.Z is enriched in the wild-type such as the 5′ end of genes.

### NRP1 co-localizes predominantly with H2A.Z depleted regions

**Fig. 4:**
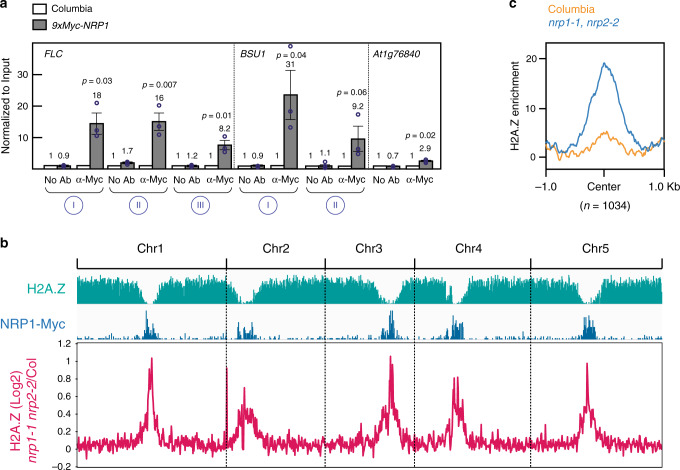
Genome-wide localization of NRP1. **a** Quantification of 9xMyc-NRP1 binding to *FLC* and *BSU1* occupancy levels in Columbia (WT) and *nrp1-1 nrp2-2* double mutant. Error bars represent standard error from three biological replicates. Two-tailed, paired Student’s *t* test was used to determine *p*-value. *At1g76840*, which has almost no reads in ChIP-Seq analysis, is shown as a control. **b** Genome-wide distribution of H2A.Z in wild-type (turquoise) and NRP1-9xMyc in wild-type (blue). In both cases, peaks were defined using Narrow Peaks. The magenta line represents differential H2A.Z occupancy between *nrp1-1 nrp2-2* and Columbia. **c** H2A.Z occupancy levels in Columbia (WT) and *nrp1-1 nrp2-2* double mutant background plotted over highly significant, likelihood ratio>1000, NRP1-9xmyc ChIP-Seq peaks. Source data underlying Fig. 4a, b are provided as a Source Data file.

To examine the distribution of the NRP proteins, we performed ChIP experiments with the *9xmyc-NRP1* complementing line. Although this approach will assess NRP1 only, we expect that NRP1 and NRP2 co-localize, given the strong interaction between NRP1 and NRP2 in vivo (Table 1 and Supplementary Fig. 4). First, we tested binding of NRP1-Myc to the putative target loci described previously; *BSU1*, *FLC*. ChIP-qPCR analyses showed a significant enrichment at all these loci compared to the negative control, (*At1g76840*), and experimental control (No antibody) (Fig. 4a). Then, we examined NRP1 distribution at the genomic level by ChIP-Seq. We found that NRP1 bound mostly, but not exclusively, to heterochromatic regions that are typically depleted of H2A.Z (Fig. 4b), this agrees with a previous study showing that *YFP-NRP1* is able to bind to the chromatin of the pericentromeric loci *Ta3*, *TSI*, and *180*bp repeats^28^. Consistently, we found that heterochromatic regions displayed the highest increase in H2A.Z occupancy in the *nrp1-1 nrp2-2* double mutant compared to wild-type, suggesting an essential role of NRP1 proteins in maintaining low H2A.Z levels around centromeres (Fig. 4b). Next, we examined the occupancy of H2A.Z at the Myc-NRP1 binding regions in both wild-type and *nrp1-1 nrp2-2* double mutant plants. When we plotted H2A.Z over highly statistically significant NRP1 peaks in the wild-type (likelihood ratio>1000), we observed only a minor peak. In contrast, in *nrp1-1 nrp2-2* double mutants, we observed a substantial H2A.Z peak at the defined NRP1 binding regions (Fig. 4c), suggesting that NRP1 is localized to areas of heterochromatin and may act to reduce H2A.Z levels at these locations.

The overall distribution pattern of both NRP1-Myc and the major gains of H2A.Z in *nrp1-1 nrp2-2* double mutants resembled that of the histone variant H2A.W, which is primarily centromeric^37^. In order to explore any possible relationship between NRP proteins and H2A.W, we examined global levels of H2A.W by western blot in Columbia and *nrp1-1 nrp2-2* double mutants and did not find any significant changes (Supplementary Fig. 5). Additionally, the H2A.W ChIP signal in the wild-type did not show any enrichment at *nrp1-1 nrp2-2* dependent H2A.Z loci (Supplementary Fig. 10). Suggesting that NRP proteins may not play a major role in H2A.W dynamics.

### NRPs do not have a critical effect on DNA methylation

We also analyzed DNA methylation patterns in *nrp1-1 nrp2-2* mutants. Whole-genome bisulfite-Seq analysis showed that methylation levels are very similar to those in the wild-type except for a very slight increase in CG and CHG methylation. This excess in methylation appears to occur mainly at transposable elements (TEs) (Supplementary Fig. 11). Moreover, H2A.Z abundance at TEs appears to be slightly higher in *nrp1-1 nrp2-2* than in the wild-type (Supplementary Fig. 11e).

In summary, the results of this study identify NRP1 and NRP2 as key factors counteracting the activity of the SWR1 complex. The opposing activities of NRP proteins dependent reduction of H2A.Z and SWR1 deposition of H2A.Z shape the genomic landscape of H2A.Z and regulate the expression of a subset of genes enriched in H2A.Z near the +1 nucleosome.

## Discussion

Plant species exhibit high levels of plasticity during development that enables them to adapt and respond in a highly plastic and dynamic fashion to fluctuating environmental conditions, which is especially important due to the sessile nature of plants. This is presumably the result of genetic factors, but recent evidence suggests that epigenetic changes could play a larger role than previously envisioned. Dynamic changes in H2A.Z occupancy have been shown to be involved in many aspects of plant biology, many of them in relation to the response to environmental cues^5,7,8,35,38^. Recently, considerable progress has been made towards the understanding of H2A.Z removal. In yeast, Inositol Requiring 80 (Ino80) has been shown to be responsible for the exchange of H2A.Z/H2B by H2A/H2B dimers^39^, and the Acidic leucine-rich nuclear phosphoprotein 32 family member E (Anp32E) has been shown to remove H2A.Z from the chromatin of mammalian cells^40–42^. However, in plants, the mechanism/s by which H2A.Z might be removed remains unclear. The role of AtINO80 regarding H2A.Z dynamics has not been clearly established; indeed, it has been shown that AtINO80 is required for H2A.Z deposition at *FLC*, *MAF4*, and *MAF5*^43^, while the possible role of ANP32E homolog in plants in regard to H2A.Z is unknown.

We have found that *nrp1-1 nrp2-2* double mutant displays an early flowering phenotype and affects the expression of genes that are essential for development such as *BSU1*, and the flowering time regulator *FLC*. Interestingly, the expression of *FLC* is known to be regulated by the histone variant H2A.Z. Moreover, we have shown that overexpression of *BSU1* is indeed caused by the disruption of *NRP1* and *2* since *BSU1* expression can be restored to nearly wild-type levels by the addition of either *NRP1* or *NRP2*-tagged lines. We have also shown functionality by showing that the *nrp1-1 nrp2-2* double mutant can rescue the wild-type phenotype of lines overexpressing *BIN2*. These data are in apparent conflict with previous studies performing transcriptomic analyses using *nrp2-1* instead of *nrp2-2* allele^28,44^. However, these differences in the phenotypes could be explained by differences in the allele strength used in each study.

Our findings also showed that the upregulation of *BSU1* that we observed in the *nrp1-1 nrp2-2* double mutant is dependent on the presence of certain levels of H2A.Z as the combination with mutations in genes coding for three of the core components of SWR1 in Arabidopsis (i.e.,- *ARP6*, *SEF*, and *PIE1*) were able to supress this upregulation. In Arabidopsis three out of twelve genes encoding for H2A and its variants are classified as H2A.Z according to phylogeny: *HTA8*, *HTA9*, and *HTA11*. Among these three genes *HTA9* and *HTA11* show much higher expression levels than *HTA8*; in fact *hta9-1 hta11-1* double mutants showed phenotypical and molecular defects typical for plants with reduced levels of H2A.Z^6^. We found that in the quadruple mutant *hta9-1 hta11-1 nrp1-1 nrp2-2*, *BSU1* expression showed no substantial difference compared to wild-type, further confirming the requirement of H2A.Z for the *BSU1* overexpression phenotype of *nrp1-1 nrp2-2*.

Our work confirmed previous reports showing that NRP1 and 2 can bind the canonical H2A^28^. Indeed, we have confirmed that NRP proteins can be found associated with H2A in vivo. But more attractively for the focus of this work, our data also showed that NRP proteins strongly interact with H2A.Z in vivo. Although Nap1 in yeast was originally thought to be a histone chaperone specific for H2A-H2B, it is worth noting that more recent studies in *Schizosaccharomyces pombe* have shown that tandem affinity purification of the histone variant H2A.Z yielded peptides of Nap1 and more interestingly the distribution of H2A.Z showed some degree of anticorrelation with that of Nap1, suggesting a role for Nap1 in H2A.Z removal^45^.

We have observed higher levels of H2A.Z in *nrp1-1 nrp2-2* compared to wild-type. This excess of H2A.Z was found to some extent in chromosome arms and gene coding regions, but it mainly localized around heterochromatic regions and centromeres, where H2A.Z is usually absent. Interestingly, overall gains of H2A.Z in *nrp1-1 nrp2-2* double mutants generally co-localized with NRP1. The fact that both H2A.Z gains in *nrp1-1 nrp2-2* and NRP1 binding sites are largely concentrated in heterochromatic regions suggests that the primary function of NRP proteins might be the regulation of H2A.Z levels at these particular regions. Alternatively, it is possible that NRP proteins also play a role in euchromatin but higher SWR1 activity and/or nucleosome turnover in the chromosome arms caused by transcription, recombination, etc… may mask the activity of NRP proteins or make more difficult the detection of NRP1 and H2A.Z gains by ChIP. In any case, these data, together with the genetic and biochemical data presented, suggests a connection between NRP proteins and reduced levels of H2A.Z.

There are at least three possible explanations for the increase in H2A.Z in *nrp1-1 nrp2-2*. One possibility is that NRP proteins simply evict H2A.Z-containing nucleosomes at discrete loci in the genome and mainly near the centromeres. A second possible scenario would be that NRP proteins would not evict H2A.Z-containing nucleosomes but instead replace H2A.Z with H2A. A third alternative could be that NRP proteins deposit H2A, H2A.W, or H2A.X, and in their absence, H2A.Z would be deposited instead by SWR1 complex, (or an unknown H2A.Z chaperone). Although further work is needed to elucidate the precise mechanism of NRP proteins, our results somewhat favor the first hypothesis for a few reasons. The H2A.Z to H2A exchange model would involve lower levels of H2A at regions of the genome in which we observed excessive levels of H2A.Z in *nrp1-1 nrp2-2*, instead, we did not observe any significant effect on the distribution of H2A in *nrp1-1 nrp2-2*. This would also be an argument against NRP proteins being responsible for H2A deposition.

The available evidence also does not support a role for NRPs in H2A.X deposition. H2A.X is thought to be mainly involved in meiosis, and its rapid accumulation is known to be an early mark of homologous recombination (HR) foci formation. The lack of NRP proteins impairs HR and the response to double-strand breaks (DSB)^30,46^, in fact, regarding HR, NRP proteins are epistatic over AtINO80, which seems to act downstream^46^. Also, NRP1 quickly accumulates after the treatment with the DSB-inducing drug CPT^30^, which suggests a role for NRP proteins in HR. However, upon loss of NRP proteins, no substantial effect is observed in the deposition of H2A.X during DNA repair at HR foci. Interestingly, in yeast and mammalian cells, the removal of H2A.Z is an essential step for proper HR during DNA repair after DSB^47,48^. Thus, it is likely that the HR phenotype observed in the *nrp1-1 nrp2-1* might be due to the inability to remove H2A.Z rather than a defect in H2A.X deposition, and it is possible that NRP proteins and AtINO80 cooperate in the removal of H2A.Z immediately after DSBs.

Although it is still unknown which protein/protein complex deposits H2A.W in Arabidopsis, it is known that H2A.W is virtually absent from coding genes^37^ while we do find NRP1 outside of the centromeres. Moreover, global levels of H2A.W appear to be unaffected in *nrp1-1 nrp2-2* double mutants.

## Methods

### Plant materials

All the lines used in this article are in Columbia-0 background. Plants were grown under standard long day conditions unless stated differently. The mutant line *nrp2-2* is Salk_205276.

### mRNA sequencing

Total RNA was purified from 100 mg of whole plant tissue (14DAG), then ground and extracted using Trizol (Invitrogen, USA). Trizol supernatant was further purified by RNeasy columns (Qiagen, Germany). The mRNA was isolated by magnetic Oligo (dT) beads, fragmentation buffer was then added. 6 bp random hexamers were used to synthesize single-stranded cDNA, double-strand cDNA then was synthesized by DNA Pol I. AMPure XP beads (Beckman Coulter, USA) were used to purify ds cDNA. 3′ Adenine and sequencing adaptors were added. AMPure XP beads were used to select the size of each segment. Finally, PCR was used to enrich the segments to obtain the cDNA library. mRNA library sequencing was done by NovoGene (Beijing, China).

### Real-time quantitative PCR

We prepared Total RNA from whole seedlings tissue (14DAG) using RNA Prep Pure Plant Kit (Tiangen, China). We used ~2 mg of RNA for the synthesis of cDNA using PrimeScript Reagent Kit with gDNA Eraser (Takara, Japan). We performed signal detection, quantification, and normalization using Quant Studio 6 proprietary Software (Life technologies, USA). For all the quantitative analyses, we performed a minimum of three biological replicates, three technical replicates each.

### Co-immunoprecipitation assays

Anti-H2A or anti-H2A.Z polyclonal antibodies were raised in rabbits using the following peptides: SGKGAKGLIMGKPSGSDKDKDKKKPIT-C/AGKGGKGLVAAKTMAANKDKDKDKKKPIS-C for H2A.Z and the peptides C-RGKTLGSGSAKKATTR and C-RGKTLGSGVAKKSTSR for H2A as an antigen (AbClonal, China). We coupled the antibody to magnetic beads using the Dynabeads antibody coupling kit (Invitrogen, USA). We ground ~2 g of 14 days old seedlings in liquid nitrogen. The we added 10 ml of IP buffer (50 mM Tris pH 7.6, 150 mM NaCl, 5 mM MgCl_2_, 10% glycerol, 0.1% NP-40, 0.5 mM DTT, 1 mM β-mercaptoethanol, 1 mM PMSF, and 1xPlant Protease Inhibitors cocktail (SIGMA, USA)). We incubated the mix on ice for 20 min, then spun at 4000 xg for 10 min at 4 °C. After that, we passed the supernatant through two layers of Miracloth (Millipore, USA). Then, we added Anti-H2A or H2A.Z antibody coupled to magnetic beads. We incubated the mix overnight at 4 °C with gentle rotation. The next morning, we washed the beads six times with IP buffer, 5 min per wash at 4 °C with gentle rotation, and then incubated at 100 °C for 15 min. We performed western blots and detection using a dilution of 1: 1000 anti-Myc 9E10 mouse monoclonal antibody (Santa Cruz Biotechnology, USA), or a dilution of 1: 1000 of anti-Flag-HRP antibody (Sigma, USA). In the case of NRP2-3xFlag and 9xMyc-NRP1 co-IP, we used anti c-Myc magnetic beads (Pierce, USA). The following steps were identical to H2A or H2A.Z co-IP.

### Protein expression analysis

Specific antibodies against proteins were used to study their expression. In this study we have used the following antibodies: Anti-Ub SC8017 (Santa Cruz Biotechnology, USA), anti-GFP N598 (MBL, Japan), anti-H3 ab1791 (Abcam, USA), anti-H2A, anti-H2A.Z, (please see Co-immunoprecipitation assays section), and anti-H2A.W^49^.

We analyzed the expression of proteins using western hybridizations. For total protein extracts, we ground about 100 mg of fresh tissue. We then transferred the powder to a tube containing 250μL of protein extraction buffer (50 mM Tris HCl, pH-7.4, 1%β-mercaptoethanol, 12% sucrose, 0.1% Triton X-100, 5 mM PMSF). We stirred and then centrifuged the mixture for 5 min at 12,000 x *g* at 4 °C. We transferred the supernatant containing the proteins to a fresh tube. We repeated this step twice. Before loading, we mixed the samples with an equal volume of 2x Laemmli loading buffer (100 mM Tris-HCl, pH = 6.8, 200 mM DTT, 4% SDS, 20% glycerol, 0.1% bromophenol blue) and then denatured the proteins by boiling the mixture for 15 min. For histone extraction we used the EpiQuik^TM^ total histone extraction kit (EPIGENTEK, USA) and followed manufacturer recommendations.

We loaded between 10-20μg of total proteins into 4–12% in the case of total protein or 12% acrylamide gels in the case of histone extracts (Invitrogen, USA) and transferred to an Immobilon-P membrane (Millipore, USA) following the instructions from the manufacturer. The antibody dilution used in each case is as follows: Anti-Ub (1:200), anti-GFP (1:2000), anti-H3 (1:5000), anti-H2A (1:1000), anti-H2A.Z (1:1000), and for anti-H2A.W we followed the dilution suggested by the authors^49^. We detected proteins using the Novex™ ECL Chemiluminescent Substrate Reagent kit (Invitrogen, USA) following the manufacturer recommendations.

### Immunopurification for mass spectrometry

We collected between 5 and 8.5 g tissue from young inflorescences from the complementing lines T_3_ *NRP2-3xFlag* or T_3_ *NRP2-9xMyc* and from Columbia plants as negative controls. Tissue was ground to a fine powder using a RETCH tissue-lyser and suspended in 20–35 mL of IP buffer, then centrifuged for 5 min at 4000 x *g* and 4 °C. The lysate was filtered through two layers of Miracloth. For *NRP2-3xFlag*, the supernatant was incubated with 250 μL FLAG M2 magnetic beads (Sigma, USA), at 4 °C for 2 h. The beads were then washed initially with 10 mL of IP buffer, then five additional times (5 min rotating at 4 °C with 1 mL IP buffer). The FLAG-IP was eluted twice with 400 µl of 250 µgmL^−1^ 3X FLAG peptides (Sigma, USA) in 1xPBS pH = 7.4 (2.7 mM KCl, 150 mM NaCl, 10 mM Na_2_HPO_4_, 1.8 mM K_2_HPO_4_), rotating for 15 min. at 4 °C. The eluted protein complexes were precipitated by the addition of 25% TCA, washed twice in ice-cold acetone and subjected to mass spectrometric analyses. For *NRP2-9xMyc* supernatant was incubated with 500 μL streptavidin-coupled magnetic beads for 1 h at 4 °C (Invitrogen, USA). Proteins were released from the streptavidin beads by 3C cleavage overnight at 4 °C. Then 3C-GST (SIGMA, USA) was removed by incubation with 300 μL of GST-coupled magnetic beads (Pierce, USA) twice rotating for 30 min. at 4 °C. The following steps were identical to *NRP2-3xFlag*. TCA precipitates were digested by the sequential addition of lys-C and trypsin proteases^50^. The digested peptides were fractionated online using reversed-phase chromatography and eluted directly into a Fusion Lumos mass spectrometer (Thermofisher, USA) where MS/MS spectra were collected^51^. Database searching was performed with the ProLuCID algorithm against a fasta protein database downloaded from The Arabidopsis Information Resource^52^. Peptide identification and protein mapping were performed using the DTASelect2 algorithm^53^. Peptide spectrum matches were filtered using a false-positive rate of less than 5% as estimated by a decoy database strategy and confident protein identifications required at least two unique peptides per protein^54^.

### Chromatin immunoprecipitation assays

For canonical H2A, H2A.Z, and Myc ChIP-Seq, we collected 2 g, 2 g, and 5 g, respectively of 14 days old seedlings grown on 1x MS plates plus 1% sucrose under long-day conditions. Plants were crosslinked in 1% Formaldehyde solution under intermittent vacuum until they became translucent, crosslinking was stopped with glycine to a final concentration of 125 mM, and then seedlings were washed in milli-Q water, blotted using Kim wipes and ground to a fine powder in a tissue lyser. The powder was then suspended in Honda Buffer (0.45 M Sucrose, 1.25% Ficoll, 2.5% Dextran, 20 mM HEPES pH7.4, 10 mM MgCl_2_, 0.5% TritonX-100, 5 mM DTT, 1 m MPMSF, and 1xPlant protease inhibitor cocktail (SIGMA, USA), incubated on ice for 20 min and filtered through two layers of Miracloth (Millipore, USA). The mix was centrifuged for 15 min at 2000 x *g* at 4 °C, the supernatant was discarded, and the pellet was again carefully resuspended into Honda Buffer. This step was repeated until pellet appeared completely white. The white pellet containing nuclei was then resuspended in Nuclei Lysis Buffer (50 mM TrisHCl pH = 8, 10 mM EDTA, 1% SDS, 1 mM PMSF, and 1xPlant protease inhibitor cocktail (SIGMA, USA), incubated on ice for 20 min. and sheared using Bioruptor Plus (Diagenode, USA) 30 s ON/OFF, Max power, 21 cycles at 4 °C. Chromatin was diluted tenfold in ChIP Dilution Buffer (167 mM NaCl, 16.7 mM TrisHCl pH8 1.2 mM EDTA, and 1.1% Triton X-100). Antibody coupled to Dynabeads was added to diluted chromatin and incubated overnight at 4 °C with gentle rotation. After that beads were recovered and washed 6 times with Washing Buffer (150 mM NaCl, 20 mM TrisHCl pH8 2 mM EDTA, 1% Triton X-100, 0,1% SDS, and 1 mM PMSF). Washed beads were then boiled for 10 min. in the presence of 10% Chelex (BioRad, USA) to reverse crosslink, then digested using 20 µg of proteinase K for an hour at 43 °C. After digestion, DNA was recovered using MinElute Spin Columns (Qiagen, Germany) following manufacturer instructions. Libraries were generated with NuGEN Ovation Ultra Low System V2 kit, according to the manufacturer’s instructions, and were sequenced on an Illumina HiSeq 4000 instrument.

In the case of Myc ChIP-qPCR everything was done in the same way except that after incubation anti-Myc antibody was pulled down using Protein A/G magnetic beads (Pierce, USA). For ChIP-qPCR at least three biological replicates were performed, three technical replicates each.

### mRNA and ChIP-seq bioinformatic analyses

ChIP-Seq reads were aligned to the reference genome (TAIR10) with Bowtie (v1.1.2), allowing only uniquely mapping reads with 0 mismatches. Duplicated reads were removed by Samtools^55^. ChIP-Seq peaks were called by MACS2^56^ (v2.1.1.) and annotated with ChIPseeker^57^. Differential peaks with likelihood ratio > 1000 were called by bdgdiff function in MACS2. Metaplots of ChIP-Seq data were plotted by deeptools (v2.5.1). To evaluate the influence of H2A.Z differential peaks to gene expression, we annotated differential peaks with ChIPseeker, and compared expression level of differential peaks’ proximal genes between Col-0 and mutants.

### Whole-genome bisulfite sequencing and analysis

Total DNA was extracted from plant (leaf) tissue (14DAG) with DNeasy kit (Qiagen, USA). Genomic DNA was converted with bisulfite treatment with EpiTect Bisulfite Kit (Qiagen, USA). Whole-genome Bisulfite Sequencing library sequencing was done by NovoGene (Beijing, China). BS-seq reads were mapped to TAIR10 reference genome by bsmap (v2.90) with allowing 2 mismatches and 1 best hit (-v 2 -w 1). Reads with three or more consecutive methylated CHH sites were considered as “unconverted” and subsequently removed. DNA methylation levels were calculated by #C/ (#C + #T). Whole-genome methylation was plotted with ViewBS (v0.1.8).

### Reporting summary

Further information on research design is available in the Nature Research Reporting Summary linked to this article.

## Supplementary information

Supplementary Information

Peer Review File

Reporting Summary

## Data Availability

Data supporting the findings of this work are available within the paper and its Supplementary Information files. A reporting summary for this article is available as a Supplementary Information file. The datasets generated and analyzed during the current study are available from the corresponding author upon request. All high-throughput sequencing data generated in this study are available at NCBI’s Gene Expression Omnibus under accession number GSE127986 [https://www.ncbi.nlm.nih.gov/geo/query/acc.cgi?acc=GSE127986]. The mass spectrometry proteomics data have been deposited to the ProteomeXchange Consortium via the PRIDE^58^ partner repository with the dataset identifier PXD019248 [https://www.ebi.ac.uk/pride/archive/projects/PXD019248]. The source data underlying Figs. 1b–d, 2, 3a, e, f, 4a, b, Table 1, as well as Supplementary Figs. 1–6, 8, 9, and 11 are provided as a Source Data file. Source data are provided with this paper.

## Source data

Source Data
