# Supplementary material for: NAP1-RELATED PROTEIN1 and 2 negatively regulate H2A.Z abundance in chromatin in Arabidopsis
Source: Nat Commun. 2020 Jun 8;11:2887. doi: 10.1038/s41467-020-16691-x (PMC7280298; doi:10.1038/s41467-020-16691-x)
Supplement: Supplementary file 1 — Supplementary Information [file 41467_2020_16691_MOESM1_ESM.pdf]

NAP1-RELATED PROTEIN1 and 2 negatively regulate H2A.Z abundance in chromatin in Arabidopsis

Wang *et al.*

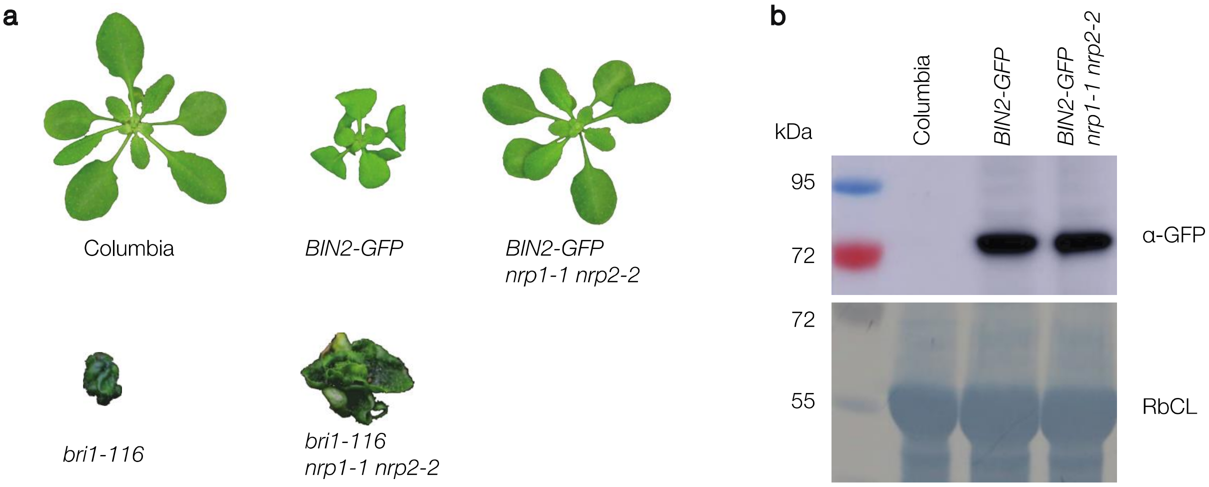

Supplementary Fig. 1. *nrp1-1 nrp2-2* double mutant suppresses the phenotype of *35S::BIN2::GFP*. a) The observed phenotype of Columbia, a *bri1-116* mutant line, a *35S::BIN2::GFP* transgenic line, and the same lines in *nrp1-1 nrp2-2* background. **b)** Western blot showing nearly identical abundance of the BIN2-GFP fusion protein in both *35S::BIN2::GFP* and *35S::BIN2::GFP nrp1-1 nrp2-2*. Amido black staining of the RUBISCO large subunit is shown as a loading control. This experiment has been performed twice producing similar results. Source data are provided as a Source Data file.

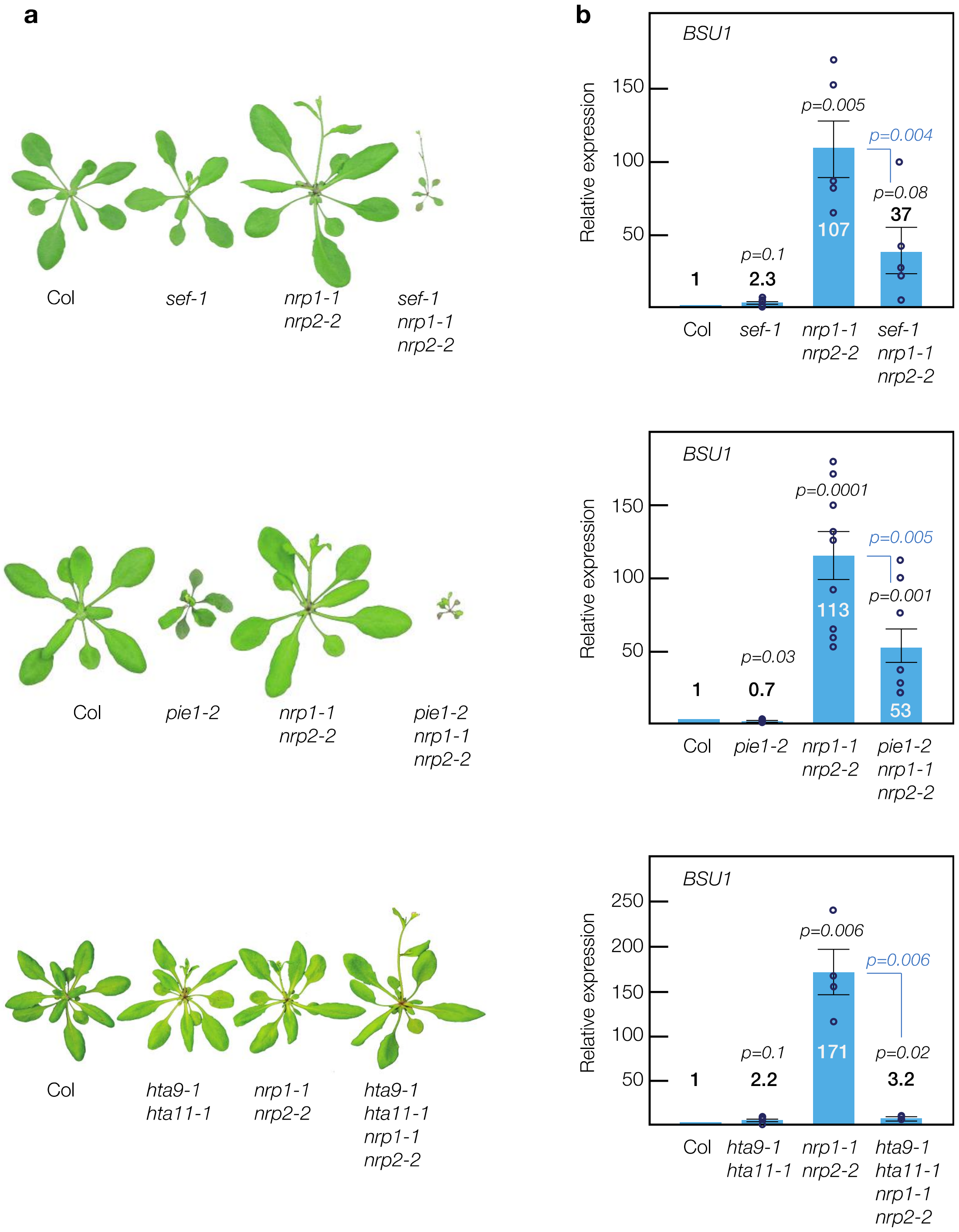

Supplementary Fig. 2. Overexpression of *BSU1* in *nrp1-1 nrp2-2* double mutants requires H2A.Z. a) Visual phenotype of the triple mutants *nrp1-1 nrp2-2 pie1-2*, *nrp1-1 nrp2-2 sef-1*, and the quadruple mutant *nrp1-1 nrp2-2 hta9-1 hta11-1*. **b)** RT-PCR showing mRNA expression levels of *BSU1* in the respective genetic backgrounds. Error bars represent standard error. *UBQ10* was used as an internal control. Two-tailed, paired Student's t-test was used to determine *p*-value. Source data are provided as a Source Data file.

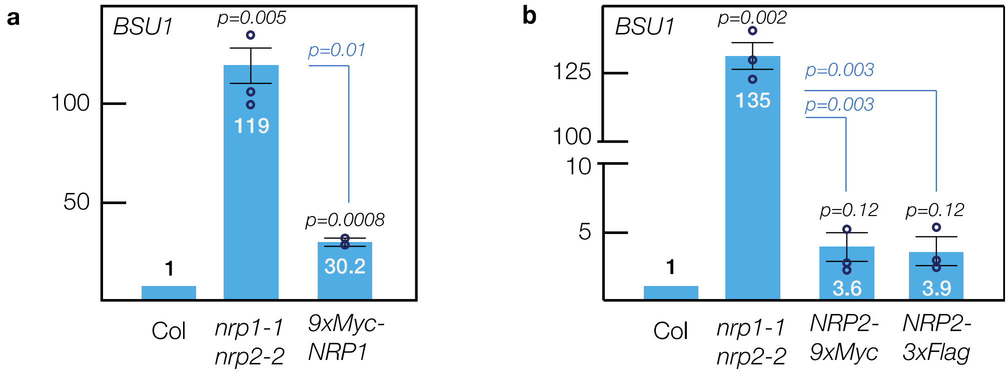

Supplementary Fig. 3. Complementation of *nrp1-1 nrp2-2* double mutants. a) RT-PCR showing mRNA expression levels of *BSU1* in Columbia, *nrp1-1 nrp2-2* double mutant, and the complementing line T_3 *nrp1-1 nrp2-2+9xMyc-NRP1*. **b)** RT-PCR showing mRNA expression levels of *BSU1* in Columbia, *nrp1-1 nrp2-2* double mutant, and the complementing lines T_3 *nrp1-1 nrp2-2+NRP2:: 9xMyc* and T_3 *nrp1-1 nrp2-2+NRP2:: 3xFlag*. Error bars represent standard error. *UBQ10* was used as an internal control. Two-tailed, paired Student's t-test was used to determine *p*-value. Source data are provided as a Source Data file.

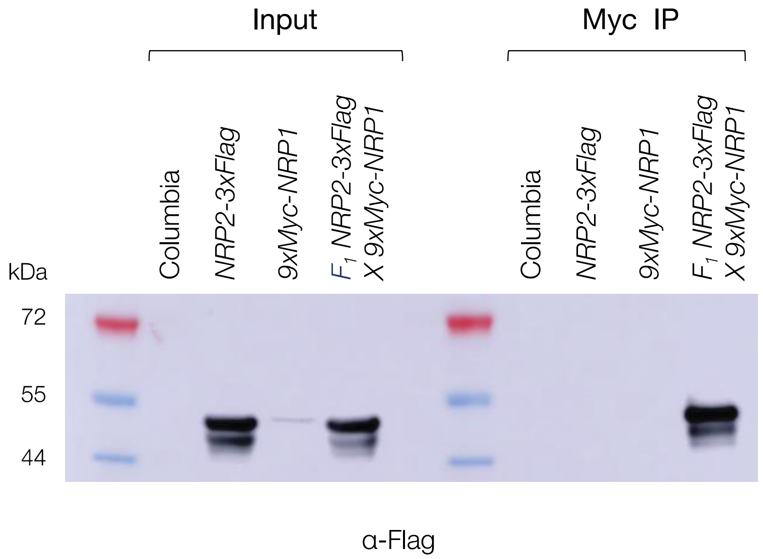

Supplementary Fig. 4. **NRP1 and NRP2 physically interact *in vivo*.** α-Myc co-immunopurification assays confirming the interaction from mass spectrometric analyses. α-Myc immunoprecipitation lanes (=Myc IP) show co-purification of NRP1 with NRP2. Protein extracts from the same plants are included to confirm the identity of the co-precipitating band (=Input). This co-immunopurification assay was repeated with similar results. Source data are provided as a Source Data file.

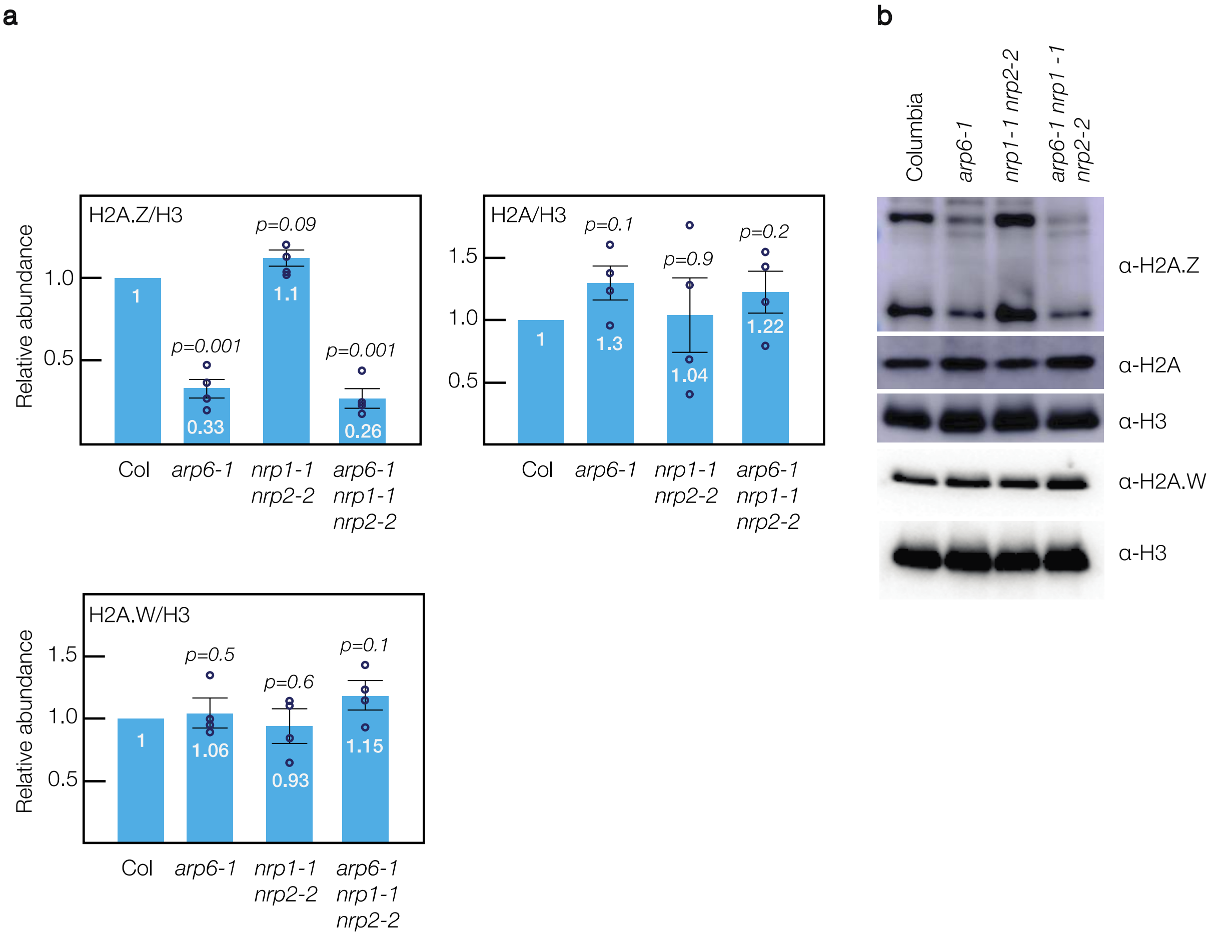

Supplementary Fig. 5. Global levels of H2A histones in *nrp1-1 nrp2-2* double mutants. a) H2A, H2A.W, and H2A.Z abundance in Columbia, *arp6-1*, *nrp1-1 nrp2-2*, and *arp6-1 nrp1-1 nrp2-2* triple mutant quantified using Image Studio Lite from four independent Western blots. In both cases, H3 was used as a control. Error bars represent standard error. **b)** Representative Western blot showing the same data. The antibody used in each case is indicated to the right of the blots. Error bars represent standard error. Two-tailed, paired Student's t-test was used to determine *p*-value. Source data are provided as a Source Data file.

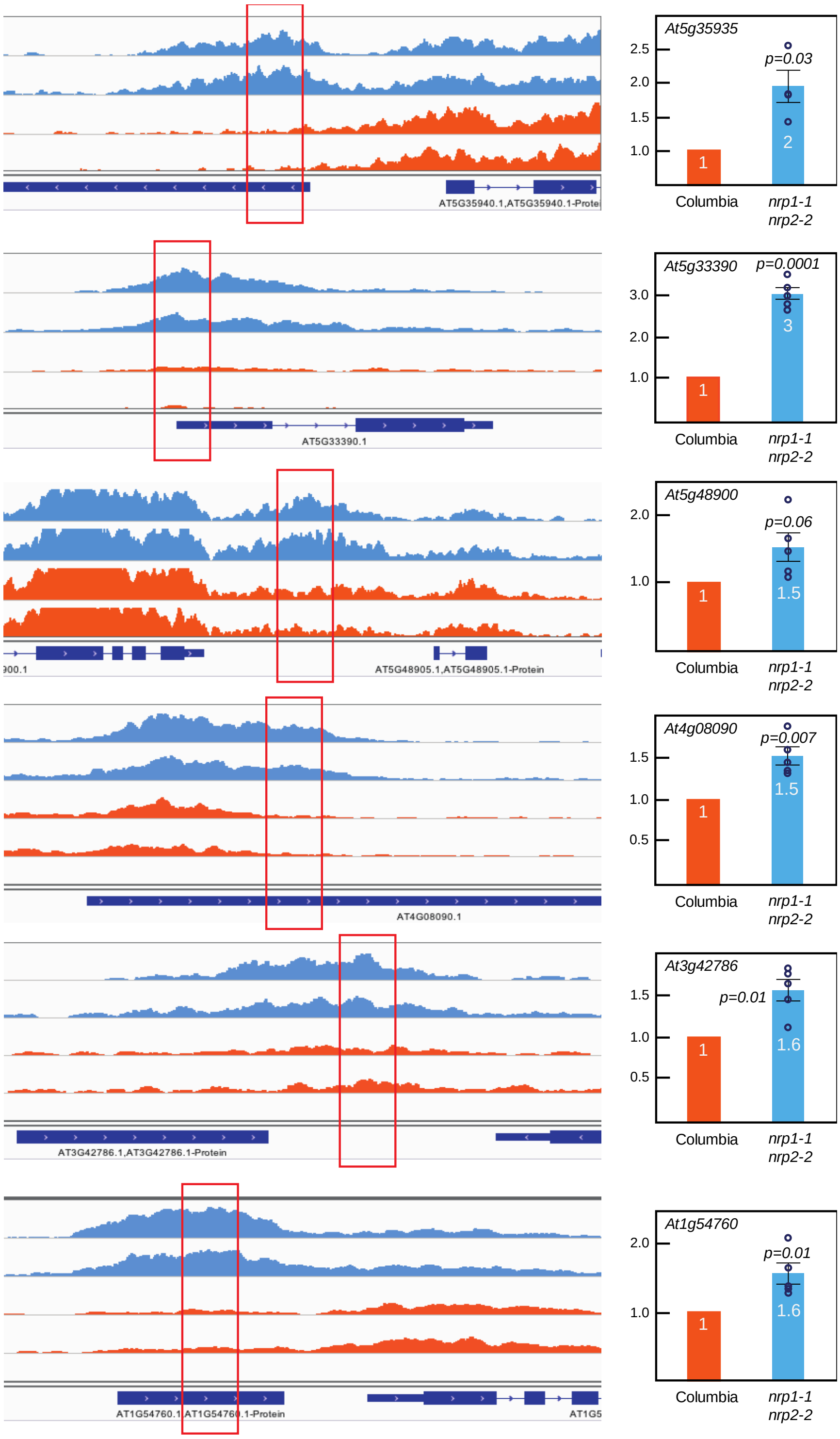

Supplementary Fig. 6. H2A.Z ChIP validation. The panels on the left show screenshots of the genomic area surrounding the validated loci. Peaks of H2A.Z ChIP over input in Columbia are represented in blue while peaks in *nrp1-1 nrp2-2* double mutants are represented in orange. A red rectangle marks the validated loci. The panels on the right show relative abundance of H2A.Z estimated by ChIP-qPCR for each locus. Estimates are the average of five independent biological replicates, each of which has three technical replicates. Two-tailed, paired Student's t-test was used to determine *p*-value. Source data are provided as a Source Data file.

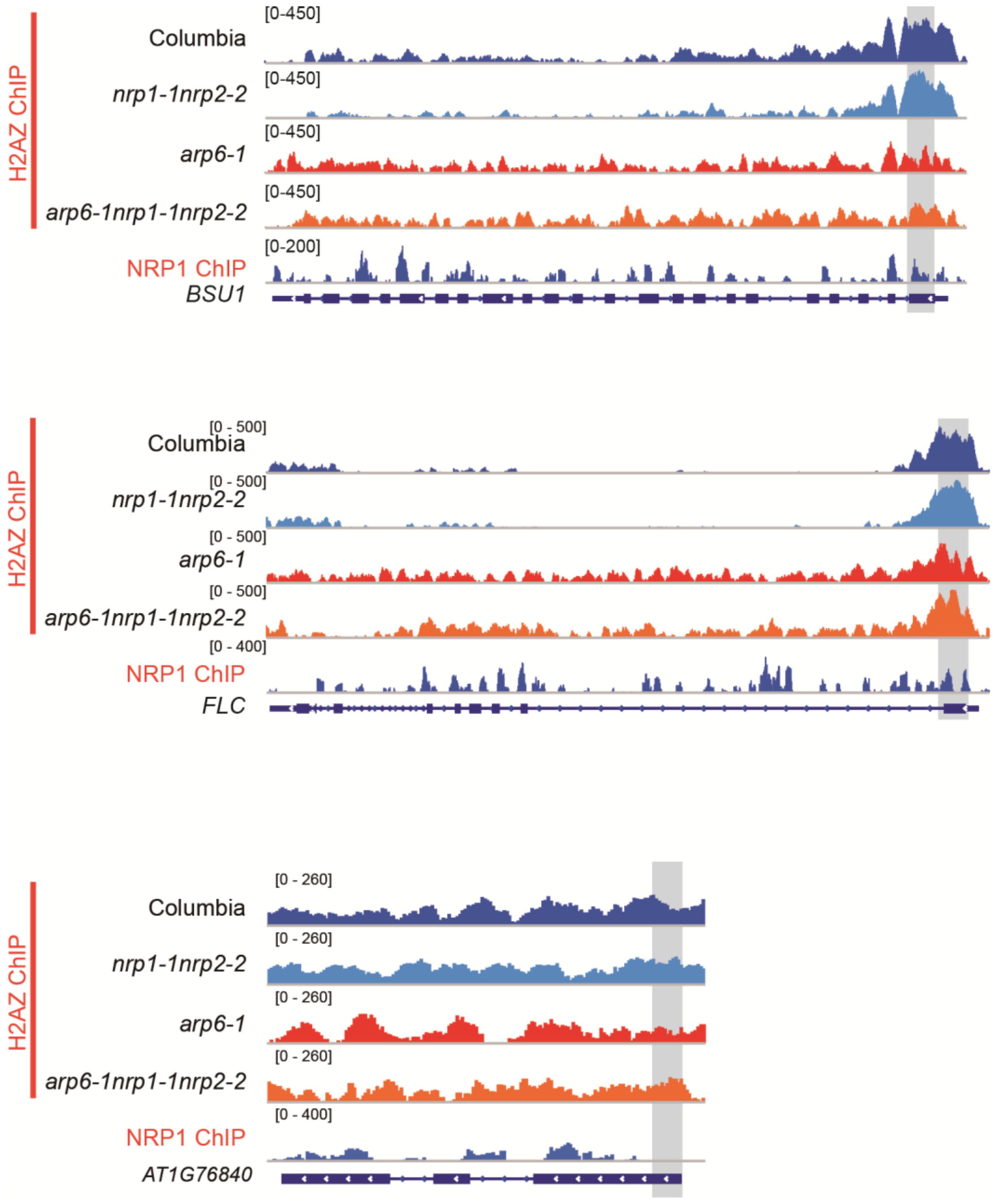

Supplementary Fig. 7. Distribution of H2A.Z and NRP1-Myc at individual loci. The panels show the distribution pattern of H2A.Z ChIP-Seq signal from Columbia, *arp6-1*, *nrp1-1 nrp2-2*, and *arp6*-1 *nrp1-1 nrp2-2*, and NRP1-Myc ChIP-Seq signal from Columbia over *BSU1*, *FLC*, and *At1g76840*.

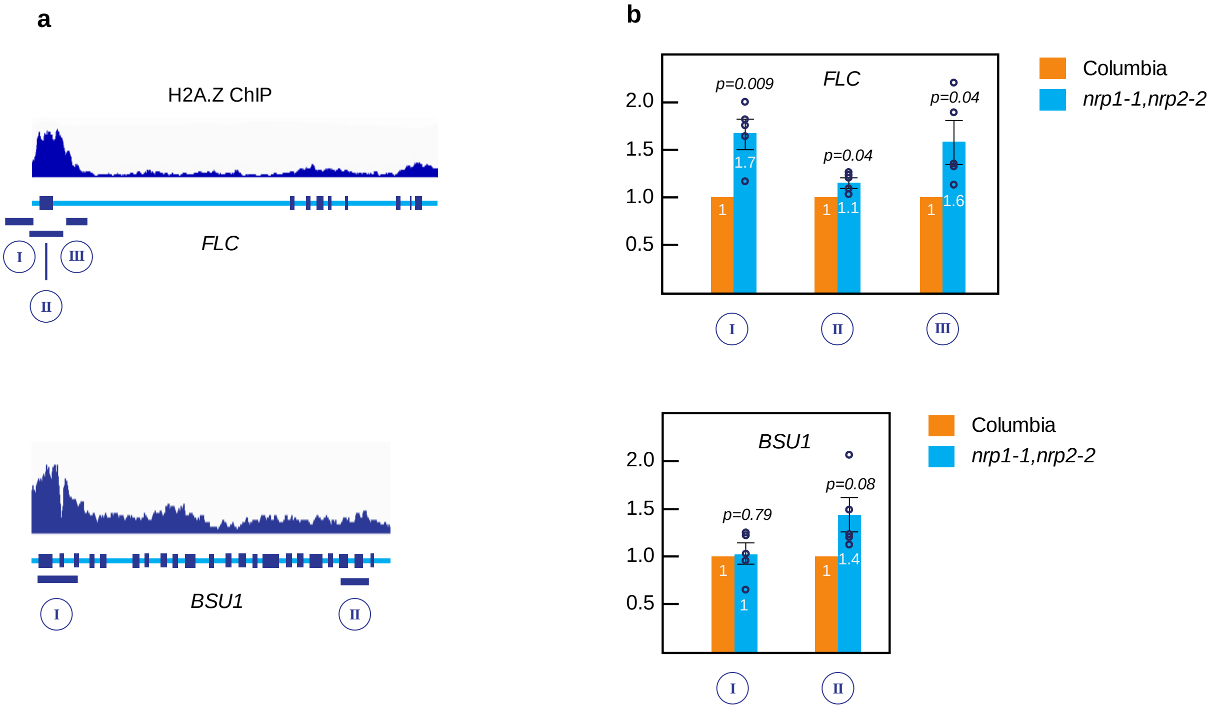

Supplementary Fig. 8. ***nrp1-1 nrp2-2*** **double mutants display overaccumulation of H3 at** ***FLC*****. a)** Schematic representation of the gene *FLC* and *BSU1*. Distribution of H2A.Z ChIP reads in wild-type across the genes is shown above. Introns and exons are shown in light blue and navy blue, respectively. **b)** The relative abundance of H3 in Columbia and *nrp1-1 nrp2-2* double mutant background measured by ChIP-qPCR at *FLC* and *BSU1* Roman numerals indicate the amplicon that was used for ChIP-qPCR analyses. ChIP signal is the average of five biological replicates, three technical replicates each, error bars indicate standard error. Two-tailed, paired Student’s t-test was used to determine *p*-value. Source data are provided as a Source Data file.

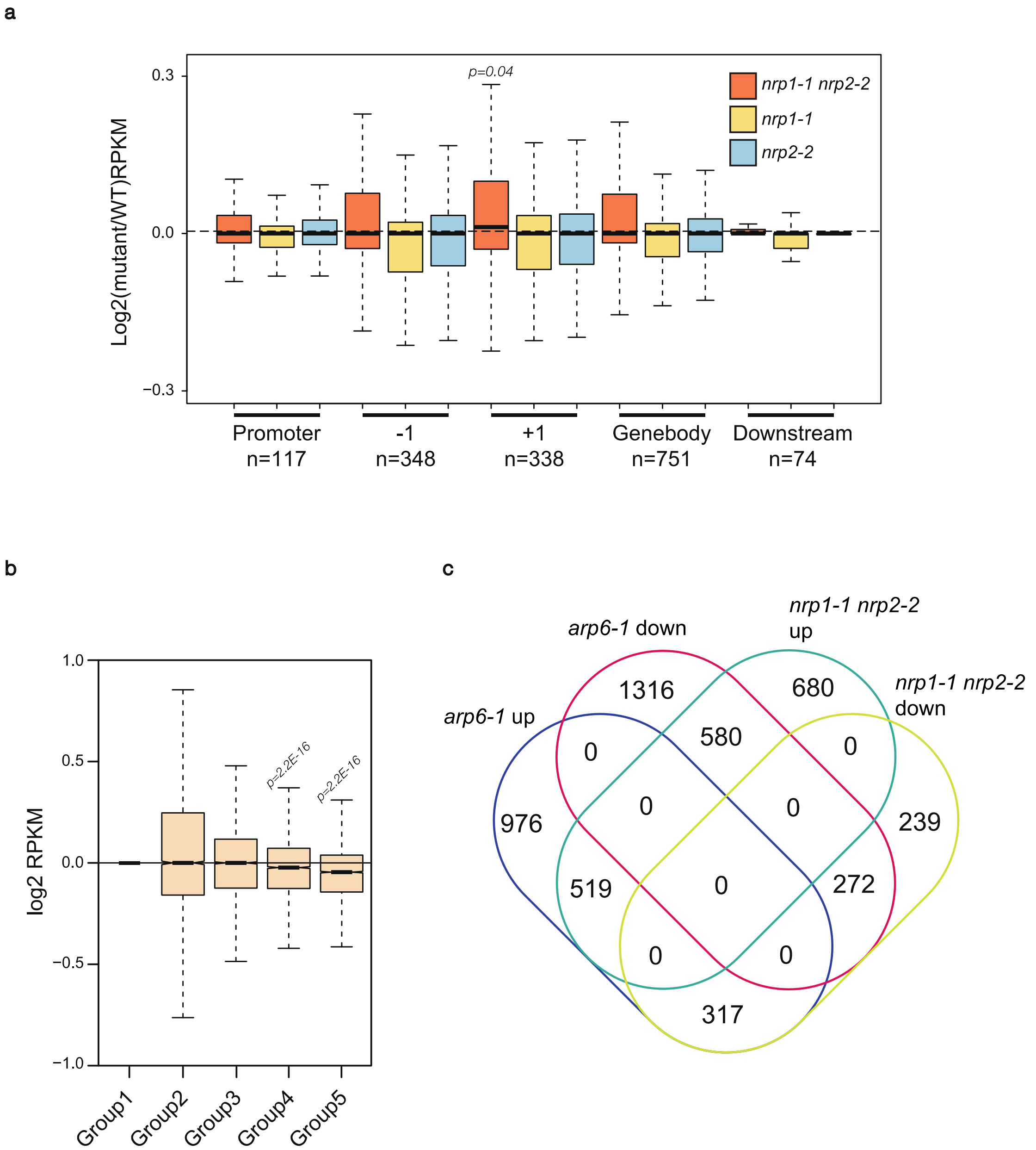

Supplementary Fig. 9. Effect of *nrp1-1 nrp2-2* on gene expression. a) Box-plot showing differential expression of *nrp1-1*, *nrp2-2*, and *nrp1-1 nrp2-2* in comparison to wild-type. For this analysis, only genes that showed a highly significant H2A.Z gain in *nrp1-1 nrp2-2* have been considered. Genes were divided according to the location of the H2A.Z peak. **b)** Box-plot showing differential expression of *nrp1-1 nrp2-2* compared to wild-type. All genes were divided according to expression level; Group1 FPKM 0-1, Group2 FPKM 1-6, Group3 FPKM 6-15, Group4 FPKM 15-30, Group5 FPKM >30. **a)** and **b)** Center lines indicate the median, boxes show the 25th (bottom) and 75th (top) percentiles; whiskers extend to the minima and maxima. Paired, two-tailed Student's t-test was used to determine *p*-value. **c)** Diagram showing the number of down-regulated and up-regulated genes (1.5-fold) in *arp6-1* and *nrp1-1 nrp2-2*. Source data are provided as a Source Data file.

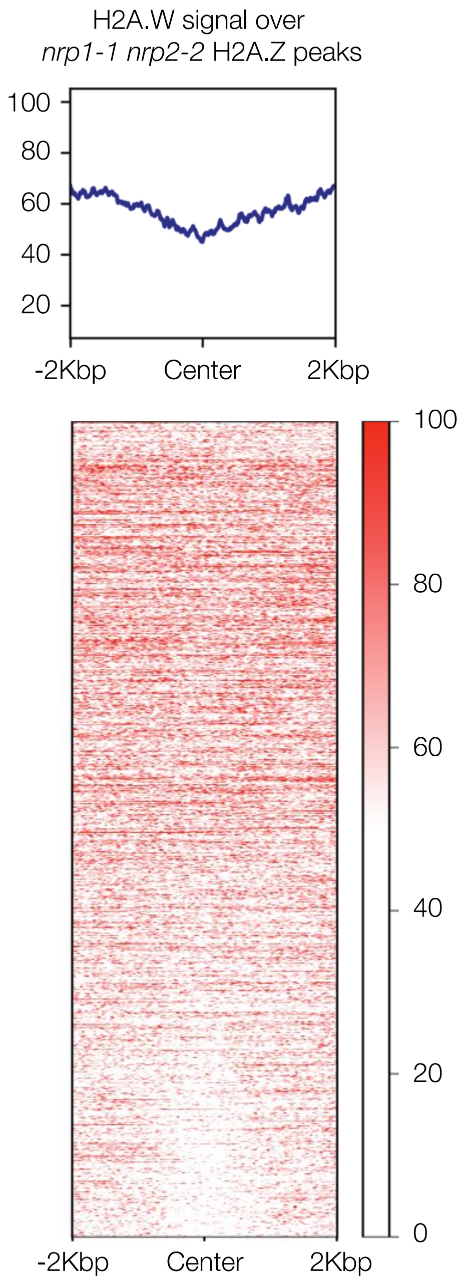

Supplementary Fig. 10. H2A.W relationship with H2A.Z dependent loci in *nrp1-1 nrp2*-2. The upper panel shows a plot depicting H2A.W levels across highly H2A.Z-enriched loci in *nrp1-1 nrp2-2* background. The lower panel shows a heatmap illustrating the same data.

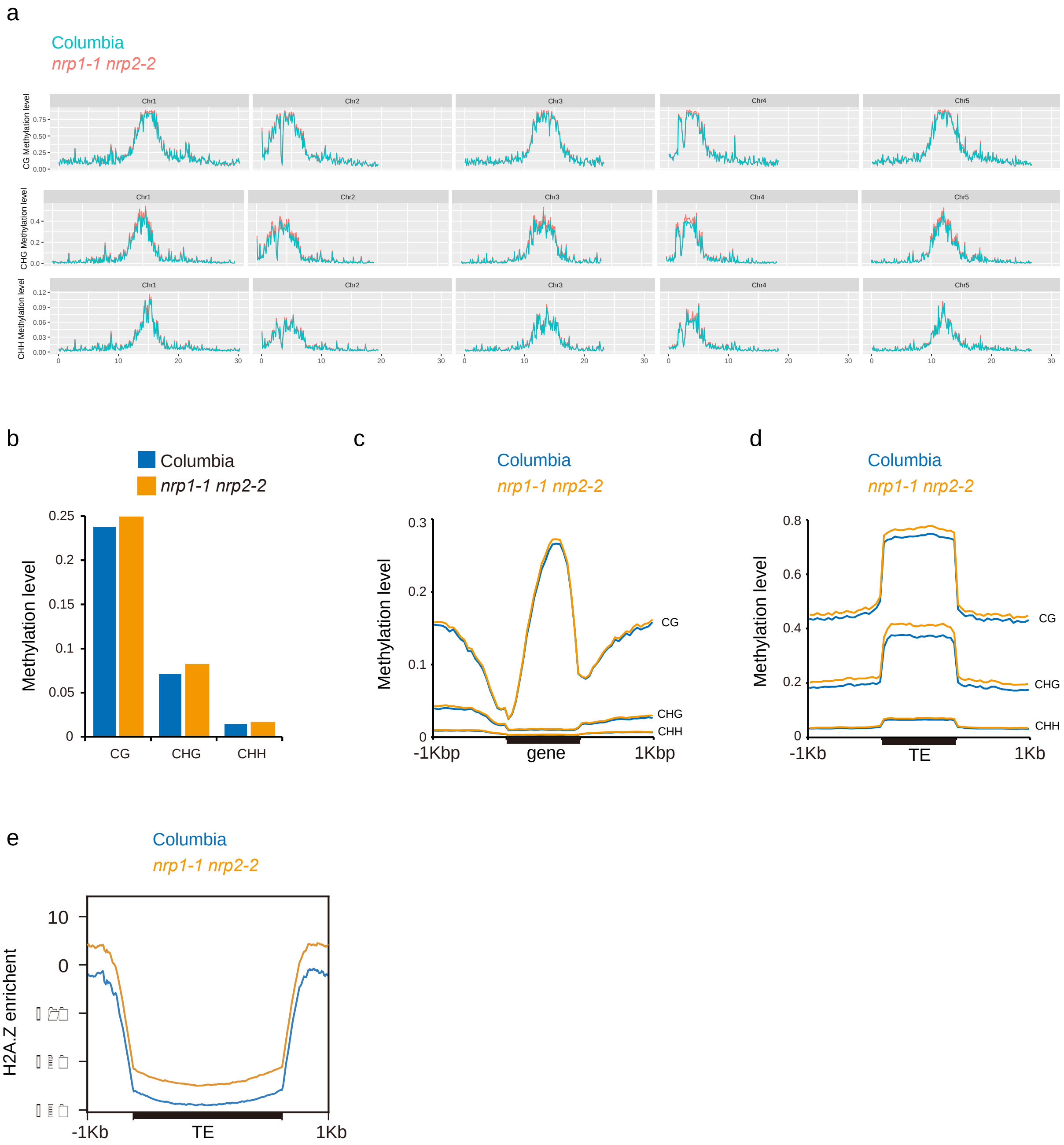

Supplementary Fig. 11. DNA methylation patterns in *nrp1-1 nrp2-2* mutants. a) The panels show the DNA methylation profile across the five chromosomes of Arabidopsis (bin=100 Kbp). CG, top panels; CHG, middle panels; CHH, bottom panels. **b)** Global methylation levels of Columbia and *nrp1-1 nrp2-2* mutants. Metagene analysis showing DNA methylation patterns of Columbia and *nrp1-1 nrp2-2* mutants over genes (*n*=28,496) **(c)** and TEs (*n*=31,189) **(d)**. **e)** H2A.Z levels plotted over TEs. Source data are provided as a Source Data file.
