# Supplementary material for: NAP1-RELATED PROTEIN1 and 2 negatively regulate H2A.Z abundance in chromatin in Arabidopsis
Source: Nat Commun. 2020 Jun 8;11:2887. doi: 10.1038/s41467-020-16691-x (PMC7280298; doi:10.1038/s41467-020-16691-x)
Supplement: Supplementary file 3 — Reporting Summary [file 41467_2020_16691_MOESM3_ESM.pdf]

nature research

Corresponding author(s): Israel Ausin
Steven E. Jacobsen

Last updated by author(s): Apr 29, 2020

Reporting Summary

Nature Research wishes to improve the reproducibility of the work that we publish. This form provides structure for consistency and transparency in reporting. For further information on Nature Research policies, see Authors & Referees and the Editorial Policy Checklist .

Statistics

For all statistical analyses, confirm that the following items are present in the figure legend, table legend, main text, or Methods section.

n/a	Confirmed	
☐	☒	The exact sample size (n) for each experimental group/condition, given as a discrete number and unit of measurement
☐	☒	A statement on whether measurements were taken from distinct samples or whether the same sample was measured repeatedly
☐	☒	The statistical test(s) used AND whether they are one- or two-sided<br>*Only common tests should be described solely by name; describe more complex techniques in the Methods section.*
☒	☐	A description of all covariates tested
☒	☐	A description of any assumptions or corrections, such as tests of normality and adjustment for multiple comparisons
☐	☒	A full description of the statistical parameters including central tendency (e.g. means) or other basic estimates (e.g. regression coefficient) AND variation (e.g. standard deviation) or associated estimates of uncertainty (e.g. confidence intervals)
☐	☒	For null hypothesis testing, the test statistic (e.g. F, t, r) with confidence intervals, effect sizes, degrees of freedom and P value noted<br>*Give P values as exact values whenever suitable.*
☒	☐	For Bayesian analysis, information on the choice of priors and Markov chain Monte Carlo settings
☒	☐	For hierarchical and complex designs, identification of the appropriate level for tests and full reporting of outcomes
☒	☐	Estimates of effect sizes (e.g. Cohen's d, Pearson's r), indicating how they were calculated

Our web collection on statistics for biologists contains articles on many of the points above.

Software and code

Policy information about availability of computer code

Data collection	no software was used.
Data analysis	ChIP-Seq reads were aligned to the reference genome (TAIR10) with Bowtie (v1.1.2), allowing only uniquely mapping reads with 0 mismatches.<br>Duplicated reads were removed by Samtools (v1.3.1).<br>ChIP-Seq peaks were called by MACS223 (v2.1.1.) and annotated with ChIPseeker.<br>Differential peaks with likelihood ratio > 1000 were called by bdgdiff function in MACS2. Metaplots of ChIP-Seq data were plotted by deeptools (v2.5.1).

For manuscripts utilizing custom algorithms or software that are central to the research but not yet described in published literature, software must be made available to editors/reviewers. We strongly encourage code deposition in a community repository (e.g. GitHub). See the Nature Research guidelines for submitting code & software for further information.

Data

Policy information about availability of data

All manuscripts must include a data availability statement. This statement should provide the following information, where applicable:

- Accession codes, unique identifiers, or web links for publicly available datasets
- A list of figures that have associated raw data
- A description of any restrictions on data availability

All high-throughput sequencing data generated in this study are available at NCBI's Gene Expression Omnibus under accession number GSE127986 [https://www.ncbi.nlm.nih.gov/geo/query/acc.cgi?acc=GSE127986]. The mass spectrometry proteomics data have been deposited to the ProteomeXchange Consortium via the PRIDE59 partner repository with the dataset identifier PXD019248 [https://www.ebi.ac.uk/pride/archive/projects/PXD019248]. Data will be made public after publication. The source data underlying Figures 1, 2, 3 and 4 as well as Supplementary Figures 1, 2, 3, 4 ,5, 6, 8, 9 and 11 are provided as a source data file. We have also used TAIR data base (www.arabidopsis.org)

Field-specific reporting

Please select the one below that is the best fit for your research. If you are not sure, read the appropriate sections before making your selection.

- [x] Life sciences
- [ ] Behavioural & social sciences
- [ ] Ecological, evolutionary & environmental sciences

For a reference copy of the document with all sections, see nature.com/documents/nr-reporting-summary-flat.pdf

Life sciences study design

All studies must disclose on these points even when the disclosure is negative.

Sample size	For flowering time experiments under long day conditions, the size of the sample was chosen based on the standard error of the average of flowering time in Columbia (wt) populations under those conditions
Data exclusions	No data exclusion in the study
Replication	For H2A, H2AZ ChIP-Seq experiment, 2 replicates of H2A, H2AZ ChIP-Seq were performed for each genotype. For NRP1 Myc, 2 replicates were preformed along with DNA input as ChIP-Seq control. For RT-PCR and ChIP-PCR three or five replicates were performed as indicated in the figure legends. Flowering time experiments and Co-IP experiments were replicated at least twice yielding similar results. IP-MassSpec was done in four replicates.
Randomization	For RNA-Seq and ChIP-Seq experiments, treatment and control samples were grown side by side, each replicate on separate MS plate.
Blinding	No blinding needed

Reporting for specific materials, systems and methods

We require information from authors about some types of materials, experimental systems and methods used in many studies. Here, indicate whether each material, system or method listed is relevant to your study. If you are not sure if a list item applies to your research, read the appropriate section before selecting a response.

Materials & experimental systems

n/a	Involved in the study	
[ ]	[x]	Antibodies
[x]	[ ]	Eukaryotic cell lines
[x]	[ ]	Palaeontology
[x]	[ ]	Animals and other organisms
[x]	[ ]	Human research participants
[x]	[ ]	Clinical data

Methods

n/a	Involved in the study	
[ ]	[x]	ChIP-seq
[x]	[ ]	Flow cytometry
[x]	[ ]	MRI-based neuroimaging

Antibodies

Antibodies used

Anti-H2A polyclonal antibodies were raised in rabbits using the peptides SGKGAKGLIMGKPSGSDKDKDKKKPIT-C / AGKGGKGLVAAKTMAANKDKDKDKKKPIS-C as an antigen (AbClonal, China). Dilution (1:1000)

Anti-H2A.Z were raised in rabbits using the peptides C-RGKTLGSGSAKKATTR and C-RGKTLGSGVAKKSTSR as an antigen (AbClonal, China)(dilution 1:1000)

Anti-H2A.W was produced in the lab of Prof. Frederic Berger kindly donated by him via Prof. Xiaofeng Cao:

Osakabe A, Lorkovic ZJ, Kobayashi W, Tachiwana H, Yelagandula R, Kurumizaka H, Berger F. Histone H2A variants confer specific properties to nucleosomes and impact on chromatin accessibility. Nucleic Acids Res. 2018 Sep 6;46(15):7675-7685. doi: 10.1093/nar/gky540. (dilution1:1000)

Anti-Ub is from Santa Cruz Biotechnology ,USA, SC8017. (Dilution 1:200)

Anti-GFP is from MBL, Japan, CodeNo 598 (dilution 1:2000)

Anti-H3 is from AbCam, USA, ab1791 (Dilution 1:5000)

Anti-Myc is 9E10 mouse monoclonal antibody from Santa Cruz Biotechnology, USA anti-Flag-HRP M2 is a8592 from sigma, USA. (Dilution 1:1000)

Validation

Validity was tested by comparison of our data with previously published data (anti-H2A and anti H2A.Z)
. In the case of commercial antibodies validation

was carried out by the manufacturer.
Anti-Ub (https://datasheets.scbt.com/sc-8017.pdf)
Anti-GFP (https://www.mblbio.com/bio/g/dtl/A/?pcd=598)
Anti-H3 (https://www.abcam.com/histone-h3-antibody-nuclear-loading-control-and-chip-grade-ab1791.html)
Anti-H2AW was produced in frederic Berger's lab and validated by them
Osakabe A, Lorkovic ZJ, Kobayashi W, Tachiwana H, Yelagandula R, Kurumizaka H, Berger F. Histone H2A variants confer specific properties to nucleosomes and impact on chromatin accessibility. Nucleic Acids Res. 2018 Sep 6;46(15):7675-7685. doi: 10.1093/nar/gky540.

ChIP-seq

Data deposition

☒ Confirm that both raw and final processed data have been deposited in a public database such as GEO.

☒ Confirm that you have deposited or provided access to graph files (e.g. BED files) for the called peaks.

Data access links
May remain private before publication.

All generated high-throughput sequencing data are available at NCBI's GEO database and are accessible via GEO Series (https://www.ncbi.nlm.nih.gov/geo) accession number GSE127986. The following secure token has been created to allow review of record GSE127986 while it remains in private status: ifehmaykvbijzed.

The mass spectrometry proteomics data have been deposited to the ProteomeXchange Consortium via the PRIDE59 partner repository with the dataset identifier PXD019248 [https://www.ebi.ac.uk/pride/archive/projects/PXD019248]

Files in database submission

arp6-1_h2az_input_r1.fq.gz arp6-1_h2az_input_r2.fq.gz arp6-1_h2az_r1.fq.gz arp6-1_h2az_r2.fq.gz col_chip_input_r1.fq.gz col_chip_input_r2.fq.gz col_chip_myc_r1.fq.gz col_chip_myc_r2.fq.gz col_h2az_input_r1.fq.gz col_h2az_input_r2.fq.gz col_h2az_r1.fq.gz col_h2az_r2.fq.gz nrp-d_h2az_input_r1.fq.gz nrp-d_h2az_input_r2.fq.gz nrp-d_h2az_r1.fq.gz nrp-d_h2az_r2.fq.gz triple_h2az_input_r1.fq.gz triple_h2az_input_r2.fq.gz triple_h2az_r1.fq.gz triple_h2az_r2.fq.gz nrp1_chip_input_r1.fq.gz nrp1_chip_input_r2.fq.gz nrp1_chip_myc_r1.fq.gz nrp1_chip_myc_r2.fq.gz
arp6-1_h2az_input_r1.bw arp6-1_h2az_input_r2.bwarp6-1_h2az_r1.bw arp6-1_h2az_r2.bw col_chip_input_r1.bw col_chip_input_r2.bw col_chip_myc_r1.bw col_chip_myc_r2.bw col_h2az_input_r1.bw col_h2az_input_r2.bw col_h2az_r1.bw col_h2az_r2.bw nrp1_chip_input_r1.bw nrp1_chip_input_r2.bw nrp1_chip_myc_r1.bw nrp1_chip_myc_r2.bw nrp-d_h2az_input_r1.bw nrp-d_h2az_input_r2.bw nrp-d_h2az_r1.bwnrp-d_h2az_r2.bw triple_h2az_input_r1.bw triple_h2az_input_r2.bw triple_h2az_r1.bw
triple_h2az_r2.bw arp6-1_h2az_r1_peaks.narrowPeak arp6-1_h2az_r2_peaks.narrowPeak col_chip_myc_r1_peaks.narrowPeak col_chip_myc_r2_peaks.narrowPeak col_h2az_r1_peaks.narrowPeak col_h2az_r2_peaks.narrowPeak nrp1_chip_myc_r1_peaks.narrowPeak nrp1_chip_myc_r2_peaks.narrowPeak nrp-d_h2az_r1_peaks.narrowPeak nrp-d_h2az_r2_peaks.narrowPeak triple_h2az_r1_peaks.narrowPeak triple_h2az_r2_peaks.narrowPeak

Genome browser session
(e.g. UCSC)

The following secure token has been created to allow review of record GSE127986 while it remains in private status: ifehmaykvbijzed.

Methodology

Replicates

For H2AZ ChIP-Seq experiment, 2 replicates of H2AZ ChIP-Seq were performed for each genotype. For nrp1 myc, 2 replicates were preformed along with DNA input as ChIP-Seq control.

Sequencing depth

col_h2az_input_r1, 36075590, 26335483, 50bp, single end col_h2az_input_r2, 39902703, 26247624, 50bp, single end col_h2az_r1, 46548040, 39606589, 50bp, single end col_h2az_r2, 44993170, 38414008, 50bp, single end arp6-1_h2az_input_r1, 33508906, 24872806, 50bp, single end arp6-1_h2az_input_r2, 47761946, 30260729, 50bp, single end arp6-1_h2az_r1, 25990816, 11353009, 50bp, single end arp6-1_h2az_r2, 32396392, 18885685, 50bp, single end nrp-d_h2az_input_r1, 42824830, 30919414, 50bp, single end nrp-d_h2az_input_r2, 38028156, 16211734, 50bp, single end nrp-d_h2az_r1, 44395923, 35996986, 50bp, single end nrp-d_h2az_r2, 39136939, 33578658, 50bp, single end triple_h2az_input_r1, 27646542, 16807303, 50bp, single end triple_h2az_input_r2, 28463313, 11606245, 50bp, single end triple_h2az_r1, 16645069, 9516339, 50bp, single end triple_h2az_r2, 28288841, 15357466, 50bp, single end col_chip_input_r1, 41270038, 29200851, 50bp, single end col_chip_input_r2, 46417675, 31949134, 50bp, single end col_chip_myc_r1, 24571401, 6213133, 50bp, single end col_chip_myc_r2, 28473981, 6948223, 50bp, single end nrp1_chip_input_r1, 33509297, 24340310, 50bp, single end nrp1_chip_input_r2, 37781394, 26672539, 50bp, single end nrp1_chip_myc_r1, 26835560, 13468742, 50bp, single end nrp1_chip_myc_r2, 30159318, 14607952, 50bp, single end

Antibodies

Anti-H2A polyclonal antibodies were raised in rabbits using the peptides SGKGAKGLIMGKPSGSDKDKDKKKPIT-C / AGKGGKGLVAAKTMAANKDKDKDKKKPIS-C as an antigen (AbClonal, China)

anti-H2A.Z were raised in rabbits using the peptides C-RGKTLGSGSAKKATTR and C-RGKTLGSGVAKKSTSR as an antigen (AbClonal, China)

anti-Myc is 9E10 mouse monoclonal antibody from Santa Cruz Biotechnology, USA

Peak calling parameters

ChIP-Seq fastq reads were aligned to the TAIR10 reference genome with Bowtie (v1.1.2) using the following parameters, -v 0 -S -m 1. All ChIP-Seq enriched peaks were called by callpeak function in MACS2 (v2.1.1.) with the parameters, -g 1.3e8 –q 0.01 –extsize 147.

Data quality

All identified peaks in the study were called with a qval threshold of 0.01 (FDR 1%).

Software

Bowtie (v1.1.2), Samtools(v1.3.1), MACS223 (v2.1.1), ChIPseeker (v1.14.2), deeptools (v2.5.1), IGV(V 3.0_beta), RStudio (V 1.0.136)
