# Supplementary material for: NAP1-RELATED PROTEIN1 and 2 negatively regulate H2A.Z abundance in chromatin in Arabidopsis
Source: Nat Commun. 2020 Jun 8;11:2887. doi: 10.1038/s41467-020-16691-x (PMC7280298; doi:10.1038/s41467-020-16691-x)
Supplement: Supplementary file 2 — Peer Review File [file 41467_2020_16691_MOESM2_ESM.pdf]

Reviewers' comments:

Reviewer #1 (Remarks to the Author):

Overall the authors provide a nice combination of genetic and epigenetic analysis to characterize the NRP double mutant (npr1/npr2) impact on Arabidopsis growth, the role for the NRPs in H2AZ disruption and interaction with SWI/SNF complex, and additional genetic analysis to support the role for the H2AZ misdistribution in these growth phenotypes. I felt the experimental design, results and analysis were solid, and for the most part support the claims except for one specific examples I will discuss below.

Figure 3 provides a nice analysis of the npr1/npr2 impact on H2AZ but I feel it could be improved with a change in the panel order. One particular change I felt could be helpful was to begin Fig 3 with the ChIP-seq and not the ChIP-PCR analysis of the specific loci. I do understand that the ChIP PCR related directly to the preceding analysis of loci impacting growth phenotypes, but as one of the main claims is the role of NRP on genome-wide H2AZ distribution, leading with the ChIP-seq made more sense to me. This could then be followed with showing the specific examples of BSU1,FWA etc. I feel that these individual loci are more of a confirmation/validation that the global disruption of the H2AZ impacts specific pathways by misregulation of these previously identified genes. I think the structural change would also help make the order of the Fig 3 panels more streamline as it currently jumps all of the place as you move from panel-to-panel (ex Fig3c to 3d). Also I feel that the genome-wide ChIP-seq data is less susceptible to locus-specific primer/PCR properties and the selection of genomic locations to sample, and as such provides a much clearer, global picture of what's going on before addressing individual loci.

Figure 3d does not appear to be correctly described in the body of the paper. In the text it states that there is higher H2AZ in nrp1/nrp2 at the n=21K H2AZ peaks in wild type. In Fig 3 image and caption though it states that these are n=1700 sites dependent on the nrp1/nrp2 mutant. As the increase in H2AZ at heterochromatin sites is a major conclusion of the paper (included in abstract) this should be corrected (or clarified if I just misread it). More generally, this increase in H2AZ at heterochromatin loci is a major finding, but is not described in much detail in Results section. It may be helpful therefore to expand on this finding more when discussing Fig 3d.

In terms of the ChIP-seq and ChIP-PCR comparison, why did they not show a comparison of the ChIP-seq of the WT, nrp1/nrp2, apr6 etc at the FWA, BSU1 loci in Fig 3 and/or in a supplemental figure. Did they not see an obvious difference in the H2AZ levels at these loci between Col and this mutants?

Line 31-32: The authors state that "little is known about the removal of H2AZ". The use of the word "removal" suggests that a mechanism is shown to support a role for the NRP proteins in this removal, though no direct evidence of this is provided. I realize the authors aren't explicitly stating this to be the case, but I feel that referring to H2AZ "removal" in the abstract is somewhat misleading as the results do not directly support this conclusion. Perhaps something like " little is know about the genes that impact H2AZ genome-wide distribution", or some other phrasing the doesn't imply a mechanism would be better.

Smaller issues-
Figure 3a: What is the pile up of reads above the gene model. I'm guessing it may be the H2AZ ChIP-seq data, but it's not mentioned in the figure caption.

Line 180: The text describes ChIP followed by RT-PCR. Is the RT here referring to "real time PCR"? I would generally think of RT-PCR referring to reverse transcriptase PCR it would be better to change this acronym to make it more clear.
Fig 1B caption has an exclamation mark "!" that I'm guessing is typo.

A few word choices I found a bit unusual such as using “simultaneous mutations” in a several places in the text instead of “double mutant”. Also, on line 128 the the authors state that the APR6 mutant “synergised” with nrp1/nrp2 mutant to impact the phenotype. I feel like saying that the nrp1/nrp2 mutant “suppressed” the apr6 mutant phenotype might be better.

Reviewer #2 (Remarks to the Author):

The manuscript by Wang and coauthors describes an analysis of the role of NRP1 and NRP2 in H2A.Z homeostasis in Arabidopsis. Most significantly, they show that NRP1 and 2 interact with H2A.Z by IP and that loss of both NRP1 and NRP2 results in excess accumulation of H2A.Z in chromatin, particularly at heterochromatic regions where the variant is not normally found. While the authors have not yet defined the underlying mechanism, this is a very exciting finding. Overall, this study is well executed and the claims are supported by the data presented. Below I highlight my relatively minor criticisms of the manuscript:

1. The title suggests that NRP1 and 2 specifically counteract the activity of SWR1, but this could be misleading. As the authors discuss toward the end of the paper, a variety of possible mechanisms exist, most of which have nothing to do with SWR1 activity, per se.
It would be fairer to say that NRP1 and NRP2 negatively regulate H2A.Z abundance in chromatin.

2. The authors state that little is known about H2A.Z removal from chromatin, overlooking the widely known phenomenon that the INO80 remodeler can perform the opposite reaction of SWR1. This should be discussed. Is it possible that the NRPs are needed for this activity of INO80? Also, it is worth pointing out that a previous report suggested that in S. pombe, NAP1 itself might be involved in H2A.Z removal (Buchanan et al. [2009} PLoS Genetics. e1000726).

3. Figures 3C and D show that there is generally more H2A.Z in chromatin in nrp1/2 doubles compared to WT and that there seems to be a slight increase in H2A.Z in chromatin in nrp1/2/arp6 compared to arp6. While the changes appear robust, it should be noted that the data are not properly normalized for nucleosome occupancy, given that the analysis is of H2A.Z/input, rather than H2A.Z/H3 ChIP. In fact, supplemental Figure 6 indicates an increase in nucleosome occupancy at FLC in the nrp1/2 double.

4. In Figure 1, there is a large difference in relative FLC expression between panels C and D. How can this be explained?

Minor points:
1. Line 45 – Nucleosomes do not contain an H2A/H2B tetramer, but rather two H2A/H2B dimers.

2. Line 70 refers to the role of MBD9 in targeting the SWR1 complex, but only one of the two papers reporting this is cited.

Reviewer #3 (Remarks to the Author):

The work of Wang et al. presents evidence that the NRP1 and 2 proteins cause a decrease of histone variant H2A.Z on chromatin. In general, this is an important study as it provides some clues about how H2A.Z histone variant is removed in Arabidopsis, which has remained elusive. However, some of

the conclusions of this manuscript are not well founded and, in my opinion, require additional work or alterations to the authors interpretations. In particular, I found that the most striking result of the work is that the increased levels of H2A.Z in nrp1-1nrp2-2 as well as the localization of NRP1 mainly occurs at heterochromatic regions; however, in my opinion this important point is under-investigated. Instead, there is a lot of work focused on four genes which results are sometimes confusing. Also, I miss a detailed transcriptome analysis of the mutants indicating which are the differential expressed genes.

Below I outline each of my concerns with this paper:

1- Figure 1- In double nrp1-1nrp2-2, the decrease in FLC relative levels found in Fig 1c is not as drastic as in Fig 1d. Are those plants grown under the same conditions?

1- Legend Fig. 1b- there is a symbol (!) that should be removed.

3- Figure 1- Are qRT-PCR data normalized to an internal control? Please, if affirmative, indicate which one (ACT2 or any other).

4- The authors indicate that they performed RNA-Seq in Columbia, nrp1-1, nrp2-2, and the nrp1-1nrp2-2 double mutant. However, they don't present any list of up- and down-regulated genes in the different mutants, which I think is an important piece of information.

5- I would recommend including a comparison of nrp1-1nrp2-2 transcriptome to that of WT and to the one of at least one swr1 mutant, like arp6 or pie (or both, there are transcriptome data available for these and other swr1 mutants). This will help to assess the overall impact of the loss of NRP1/2 function, which is difficult to evaluate considering the expression of only 4 genes (FLC, MAF4, MAF5 and BSU1).

6- It would be also important to indicate what type of genes are the ones that experience expression change (coding, non-coding, TEs). I am asking because in Supplementary Fig 8, most if not all the genes used to validate the increased levels of H2A.Z in nrp1-1nrp2-2 are TEs.

7- It is proposed that the increased levels of H2A.Z leads to overexpression of BSU1, which is consistent with the increased levels of H2A.Z found in nrp1-1nrp2-2 when measured by ChIP-PCR and with the expression of the gene detected in the mutant. According to Fig. 3a, the levels of H2A.Z at BSU1 seem to be high and extended across the gene body in WT, which is consistent with its low expression levels (Fig 1), however, the expression of the gene is not altered in arp6 mutant in which the levels of H2A.Z are reduced, suggesting that the repression of the gene is not affected by a decrease in H2A.Z. The authors indicate that NRP1/2 could play a specific role at low expressed genes in which the increase of H2A.Z occupancy in nrp1-1nrp2-2 takes place immediately after the TSS (+1 nucleosome), but not other regions, is this the case of BSU1? I would recommend to include screenshots of the genomic area of BSU1 showing H2A.Z levels in nrp1-1nrp2-2, arp6 and in the triple mutant.

8- In the case of FLC, MAF4 and 5 the effect of nrp1-1nrp2-2 mutation is puzzling.
- In nrp1-1 nrp2-2 mutant, as in arp6-1, FLC expression levels are significantly decreased compared to WT and its expression is not recovered in the triple mutant, indicating that the loss and the gain of H2A.Z levels similarly impact gene expression, which is not consistent with a requirement of H2A.Z for FLC expression. In fact, in nrp1-1 nrp2-2 the levels of H2A.Z seem to be similar to WT and the gene is down regulated. The authors, in an attempt to find a possible explanation for this, propose that the high levels of H3 found at the first intron of FLC in nrp1-1 nrp2-2 double mutant could indicate a higher degree of chromatin compaction that may explain the negative effect of H2A.Z levels in nrp1-1nrp2-2. Is this something that only happens in FLC? Also, as for the case of BSU1, I recommend to include screenshots of the H2A.Z levels at FLC in nrp1-1nrp2-2, arp6 and triple mutant.

- On the other hand, MAF4 and 5 expression levels are recovered in the triple mutant, but what happens with H2A.Z levels, are they recovered? Again, please include screenshots of the H2A.Z levels at MAF4 and 5 in nrp1-1nrp2-2, arp6 and triple mutant.

9- According to previous data, NRP1 can interact with all canonical histones, and here the authors clearly show an in vivo interaction with H2A.Z. I wonder whether the authors found H2A and/or H2A.Z among the peptides detected in IP-MS experiment using NRP2-Flag and NRP2-Myc. You mentioned that only unique peptides across all replicates are shown, however, if the NRP proteins interact with H2A and H2A.Z, an enrichment of peptides corresponding to these histones should be detected compared to WT Col control. Also, despite the authors found a consistent enrichment of At4g31620 peptides in all IPs, they did not comment anything about this B3 factor. Is there any information about its possible role? Could this interaction fit with NRP function?

10- Fig 1f- I would recommend to include the heatmap of H2A.Z peaks in WT together with the one in arp6 and the triple mutant.

11- ;Metagene plot in nrp1-1 nrp2-2 show an increase of H2A.Z along the entire gene, which does not fit with the proposal that the increase in H2A.Z occupancy in nrp1-1nrp2-2 occurs in the region immediately after the TSS (+1 nucleosome), but not other regions (Line 207 to 216). It would be interesting to include the metagene plots of H2A.Z in arp6 and the triple mutant.

12- It is surprising that the levels of H2A do not change in nrp1-1nrp2-2. If NRP1/2 mitigate the excessive deposition of H2A.Z, thus balancing H2A/H2A.Z levels, a decrease in H2A should be expected in nrp1-1nrp2-2. If NRP1 is mostly bound to heterochromatic regions and H2A.Z increased levels are mostly found at these regions (which I consider the most interesting finding of the manuscript), it might be possible that it acts mitigating the incorporation of H2A.Z at H2A.W domains. It would be interesting to investigate the levels of H2A.W in nrp1-1nrp2-2 or at least whether the regions with increased H2A.Z levels are normally occupied by H2A.W (genome wide localization data of H2A.W are already available).

13- I recommend to include also screenshots of NRP1 localization at FLC, MAF4, 5 and BSU1.

14- The authors indicate that the increased occupancy in nrp1-1nrp2-2 tends to occur in low expressed genes (Group 1, 2 and 3). Which genes are included in this groups? How many genes of these groups are upregulated? Are they TEs? The validated genes are TEs (At1g54760, At4g08090, At5g35935) or close to TEs (At3g42786, At5g33390) genes. Are they group 1 genes? In which group are included FLC, MAF4/5 and BSU1?

15- In the discussion, the authors contemplate several possibilities to explain the increased levels of H2A.Z in nrp1-1 nrp2-2. The authors favor the hypothesis that NRP1/2 is involved in eviction of H2A.Z, which I consider plausible. In any case, an increase of H2A.Z in the double mutant implies that another H2A is removed. According to the results, it seems that it is not canonical H2A as its levels are not altered in nrp1-1 nrp2-2. In supplementary Figure 6, the authors checked by WB the global levels of H2A.Z in different mutants, it would be interesting to include H2A.X and H2A.W in the analysis. An intriguing possibility that in my opinion deserves to be investigated is that NRP1/2 evict H2A.Z from the regions where H2A.W should be incorporated.

First, we would like to thank all the reviewers for their suggestions and their useful comments that have greatly contributed to improving our manuscript.

Reviewer #1 (Remarks to the Author):

Overall the authors provide a nice combination of genetic and epigenetic analysis to characterize the NRP double mutant (npr1/npr2) impact on Arabidopsis growth, the role for the NRPs in H2AZ disruption and interaction with SWI/SNF complex, and additional genetic analysis to support the role for the H2AZ misdistribution in these growth phenotypes. I felt the experimental design, results and analysis were solid, and for the most part support the claims except for one specific examples I will discuss below.

Figure 3 provides a nice analysis of the npr1/npr2 impact on H2AZ but I feel it could be improved with a change in the panel order. One particular change I felt could be helpful was to begin Fig 3 with the ChIP-seq and not the ChIP-PCR analysis of the specific loci. I do understand that the ChIP PCR related directly to the preceding analysis of loci impacting growth phenotypes, but as one of the main claims is the role of NRP on genome-wide H2AZ distribution, leading with the ChIP-seq made more sense to me. Tnhis could then be followed with showing the specific examples of BSU1,FWA etc. I feel that these individual loci are more of a confirmation/validation that the global disruption of the H2AZ impacts specific pathways by misregulation of these previously identified genes. I think the structural change would also help make the order of the Fig 3 panels more streamline as it currently jumps all of the place as you move from panel-to-panel (ex Fig3c to 3d). Also I feel that the genome-wide ChIP-seq data is less susceptible to locus-specific primer/PCR properties and the selection of genomic locations to sample, and as such provides a much clearer, global picture of what's going on before addressing individual loci.

Thank you for these positive comments and for the suggestion. We have changed Figure 3 as suggested and we have also changed the order in the main text.

Figure 3d does not appear to be correctly described in the body of the paper. In the text it states that there is higher H2AZ in nrp1/nrp2 at the n=21K H2AZ peaks in wild type. In Fig 3 image and caption though it states that these are n=1700 sites dependent on the nrp1/nrp2 mutant.

We are sorry for the mistake, it has now been corrected in the main text.

"We examined H2A.Z peaks defined in wild-type (n=21,024) and observed significantly higher average occupancies of H2A.Z in the nrp1-1 nrp2-2 double mutant compared to wild-type ***(Fig. 3a)****."*

"Additionally, we found 1,753 highly significantly enriched H2A.Z peaks that were nrp1-1 nrp2-2 dependent, (p-value < 2.2e-16) ***(Fig. 3c)"***

As the increase in H2AZ at heterochromatin sites is a major conclusion of the paper (included in abstract) this should be corrected (or clarified if I just misread it). More generally, this increase in H2AZ at heterochromatin loci is a major finding, but is not described in much detail in Results section. It may be helpful therefore to expand on this finding more when discussing Fig 3d.

Thanks for the suggestion. Yes, it is also in our opinion a major finding of our work. We have now added an extra paragraph in the discussion section.

"Interestingly, overall gains of H2A.Z in nrp1-1 nrp2-2 double mutants generally co-localized with NRP1. The fact that both H2A.Z gains in nrp1-1 nrp2-2 and NRP1 binding sites are largely concentrated in heterochromatic regions suggests that the primary function of NRP proteins might be the regulation of H2A.Z levels at these particular regions. Alternatively, it is possible that NRP proteins also play a role in euchromatin but higher SWR1 activity and/or nucleosome turnover in the chromosome arms caused by transcription, recombination, etc… may mask the activity of NRP proteins or make more difficult the detection of NRP1 and H2A.Z gains by ChIP"

In terms of the ChIP-seq and ChIP-PCR comparison, why did they not show a comparison of the ChIP-seq of the WT, nrp1/nrp2, apr6 etc at the FWA, BSU1 loci in Fig 3 and/or in a supplemental figure. Did they not see an obvious difference in the H2AZ levels at these loci between Col and this mutants?

We did indeed see differences in five independent replicates of ChIP-PCR. In addition, as suggested, we now have added screenshots of *FLC*, (we guessed that is what you meant by *FWA*), *BSU1*, *MAF4* and *MAF5*. This is now Supplementary Figure 7.

Line 31-32: The authors state that "little is known about the removal of H2AZ". The use of the word "removal" suggests that a mechanism is shown to support a role for the NRP proteins in this removal, though no direct evidence of this is provided. I realize the authors aren't explicitly stating this to be the case, but I feel that referring to H2AZ "removal" in the abstract is somewhat misleading as the results do not directly support this conclusion. Perhaps something like " little is know about the genes that impact H2AZ genome-wide distribution", or some other phrasing the doesn't imply a mechanism would be better.

We agree and we have removed the term "removal" and rephrase the sentence as it follows:

"in plants little is known about how a reduction of H2A.Z levels can be achieved"

Smaller issues-
Figure 3a: What is the pile up of reads above the gene model. I'm guessing it may be the H2AZ ChIP-seq data, but it's not mentioned in the figure caption.

Thank you for pointing this out, we certainly failed to properly explain that panel. We have corrected the former figure 3a (now 3e) to this;

"e) The upper part of the panel shows the distribution of H2A.Z ChIP reads across the genes immediately below"

Line 180: The text describes ChIP followed by RT-PCR. Is the RT here referring to "real time PCR"? I would generally think of RT-PCR referring to reverse transcriptase PCR it would be better to change this acronym to make it more clear.

Thank you for noticing this, the acronym RT-PCR has been now removed

Fig 1B caption has an exclamation mark "!" that I'm guessing is typo.

Thanks, yes, it was a typo. It has been removed

A few word choices I found a bit unusual such as using “simultaneous mutations” in a several places in the text instead of “double mutant”. Also, on line 128 the the authors state that the APR6 mutant “synergised” with nrp1/nrp2 mutant to impact the phenotype. I feel like saying that the nrp1/nrp2 mutant “suppressed” the apr6 mutant phenotype might be better.

Thank you, these expressions have been changed as per reviewer suggestion.

Reviewer #2 (Remarks to the Author):

The manuscript by Wang and coauthors describes an analysis of the role of NRP1 and NRP2 in H2A.Z homeostasis in Arabidopsis. Most significantly, they show that NRP1 and 2 interact with H2A.Z by IP and that loss of both NRP1 and NRP2 results in excess accumulation of H2A.Z in chromatin, particularly at heterochromatic regions where the variant is not normally found. While the authors have not yet defined the underlying mechanism, this is a very exciting finding. Overall, this study is well executed and the claims are supported by the data presented. Below I highlight my relatively minor criticisms of the manuscript:

Thank you for these supportive comments!

1. The title suggests that NRP1 and 2 specifically counteract the activity of SWR1, but this could be misleading. As the authors discuss toward the end of the paper, a variety of possible mechanisms exist, most of which have nothing to do with SWR1 activity, per se.
It would be fairer to say that NRP1 and NRP2 negatively regulate H2A.Z abundance in chromatin.

We appreciate the suggestion and agree. The title of the manuscript has been changed to:

“*NAP1-RELATED PROTEIN1 and 2 negatively regulate H2A.Z abundance in chromatin”*

2. The authors state that little is known about H2A.Z removal from chromatin, overlooking the widely known phenomenon that the INO80 remodeler can perform the opposite reaction of SWR1. This should be discussed. Is it possible that the NRPs are needed for this activity of INO80? Also, it is worth pointing out that a previous report suggested that in S. pombe, NAP1 itself might be involved in H2A.Z removal (Buchanan et al. [2009} PLoS Genetics. e1000726).

We are sorry about the imprecision, we did not mean to overlook the work that has been done in other organisms. We now mention the role of Ino80 and Anp32E in the removal of H2A.Z. We added the following paragraph.

“Recently, considerable progress has been made towards the understanding of H2A.Z removal. In yeast, Inositol Requiring 80 (Ino80) has been shown to be responsible for the exchange of H2A.Z/H2B by H2A/H2B dimers[39], and the Acidic leucine-rich nuclear phosphoprotein 32 family member E (Anp32E) has been shown to remove H2A.Z from the chromatin of mammalian cells[40, 41, 42]. However, in plants the mechanism/s by which H2A.Z might be removed remains unclear. The role of AtINO80 regarding H2A.Z dynamics has not been clearly established; indeed, it has been shown that AtINO80 is required for H2A.Z deposition at FLC, MAF4 and MAF5[43], while the possible role of ANP32E homolog in plants in regard to H2A.Z is unknown.”

And yes, this is a good argument. Indeed, it has been recently suggested that NRP proteins and AtINO80 cooperate, at least in the context of the HR pathway following Double strand breaks (former reference 38, actual 46), and we have added the paragraph below:

"The lack of NRP proteins impairs HR and the response to double strand breaks (DSB)[30, 45], in fact, regarding HR, NRP proteins are epistatic over AtINO80, which seems to act downstream[45]. Also, NRP1 quickly accumulates after the treatment with the DSB-inducing drug CPT[30], which suggests a role for NRP proteins in HR. However, upon loss of NRP proteins, no substantial effect is observed in the deposition of H2A.X during DNA repair at HR foci. Interestingly, in yeast and mammalian cells the removal of H2A.Z is an essential step for proper HR during DNA repair after DSB[46, 47]. Thus, it is likely that the HR phenotype observed in the nrp double mutants might be due to the inability to remove H2A.Z rather than a defect in H2A.X deposition. And it is possible that NRP proteins and AtINO80 cooperate in the removal of H2A.Z immediately after DSBs".

And thank you for bringing this paper to our attention. We have added this paragraph to the discussion section:

"Although Nap1 in yeast was originally thought to be a histone chaperone specific for H2A-H2B, it is worth noting that more recent studies in Schizosaccharomyces pombe have shown that tandem affinity purification of the histone variant H2A.Z yielded peptides of Nap1 and more interestingly the distribution of H2A.Z showed some degree of anticorrelation with that of Nap1, suggesting a role for Nap1 in H2A.Z removal[45]".

3. Figures 3C and D show that there is generally more H2A.Z in chromatin in nrp1/2 doubles compared to WT and that there seems to be a slight increase in H2A.Z in chromatin in nrp1/2/arp6 compared to arp6. While the changes appear robust, it should be noted that the data are not properly normalized for nucleosome occupancy, given that the analysis is of H2A.Z/input, rather than H2A.Z/H3 ChIP. In fact, supplemental Figure 6 indicates an increase in nucleosome occupancy at FLC in the nrp1/2 double.

It is correct that H2A.Z ChIPs are normalized to input and as the reviewer points out it could be possible that the observed increase in H2A.Z might be, in part, due to higher nucleosome occupancy. This could be the case of *FLC,* although it is likely not the case of *BSU1* (Supplementary Fig. 8) where we did not see an increase in H3 occupancy. We did attempt some H3 ChIP-seq experiments, but they were unfortunately not successful, and given that the Ausin lab has recently relocated we would not be able to complete this in timely manner. In any event, we feel that whether the changes of H2A.Z abundance in *nrp1-1 nrp2-2* are generally due to a denser chromatin (which will point towards an eviction mechanism) or just more H2A.Z in place of other H2A variants (which will point towards a replacement mechanism), we think that the fact that there is an increase of H2A.Z in *nrp1-1 nrp2-2* is biologically relevant and H2A.Z as well as H2A overall patterns in Columbia are consistent with previous work, indicating that our ChIPs are working correctly. We also feel that the increases in H2A.Z we see are robust and we hope the referee is satisfied with this response.

4. In Figure 1, there is a large difference in relative FLC expression between panels C and D. How can this be explained?

We had also noticed this difference in *FLC* expression. In both cases the tissue is whole plant, (excluding roots), grown under long day photoperiod, they were collected at the same developmental stage (14DAG), same approximate time of the day and approximately the same temperature. We thought this was a response to differences in light quantity and/or quality but unfortunately, we could never properly test it since the plant chamber in which the plants from figure 3C were grown no longer exist. However, we do not feel that this difference affects the conclusions in the paper.

Minor points:

1. Line 45 – Nucleosomes do not contain an H2A/H2B tetramer, but rather two H2A/H2B dimers.

Thank you for the correction, we have fixed this mistake.

2. Line 70 refers to the role of MBD9 in targeting the SWR1 complex, but only one of the two papers reporting this is cited.

We are very sorry about this; parts of the manuscript were already written before the work of Sijacic *et al*., 2019 appeared. We have fixed this in the current version (reference 21).

Reviewer #3 (Remarks to the Author):

The work of Wang et al. presents evidence that the NRP1 and 2 proteins cause a decrease of histone variant H2A.Z on chromatin. In general, this is an important study as it provides some clues about how H2A.Z histone variant is removed in Arabidopsis, which has remained elusive. However, some of the conclusions of this manuscript are not well founded and, in my opinion, require additional work or alterations to the authors interpretations. In particular, I found that the most striking result of the work is that the increased levels of H2A.Z in nrp1-1nrp2-2 as well as the localization of NRP1 mainly occurs at heterochromatic regions; however, in my opinion this important point is under-investigated. Instead, there is a lot of work focused on four genes which results are sometimes confusing. Also, I miss a detailed transcriptome analysis of the mutants indicating which are the differential expressed genes.

Below I outline each of my concerns with this paper:

1- Figure 1- In double nrp1-1nrp2-2, the decrease in FLC relative levels found in Fig 1c is not as drastic as in Fig 1d. Are those plants grown under the same conditions?

We had also noticed this difference in *FLC* expression. In both cases the tissue is whole plant, (excluding roots), grown under long day photoperiod, they were collected at the same developmental stage (14DAG), same approximate time of the day and approximately the same temperature. We thought this was a response to differences in light quantity and/or quality but unfortunately, we could never properly test it since the plant chamber in which the plants from figure 3C were grown no longer exist. However, we do not feel that this difference affects the conclusions in the paper.

1- Legend Fig. 1b- there is a symbol (!) that should be removed.

Thank you, it has been removed.

3- Figure 1- Are qRT-PCR data normalized to an internal control? Please, if affirmative, indicate which one (ACT2 or any other).

We are sorry about the lack of information. Yes, they are normalized to *UBQ10*. We have added the information in the figure legends.

4- The authors indicate that they performed RNA-Seq in Columbia, nrp1-1, nrp2-2, and the nrp1-1nrp2-2 double mutant. However, they don't present any list of up- and down-regulated genes in the different mutants, which I think is an important piece of information.

Thank you for the suggestion, and we added a supplementary file containing the complete analysis of the RNA-Seq data including a list of up-regulated and down-regulated genes.

5- I would recommend including a comparison of nrp1-1nrp2-2 transcriptome to that of WT and to the one of at least one swr1 mutant, like arp6 or pie (or both, there are transcriptome data available for these and other swr1 mutants). This will help to assess the overall impact of the loss of NRP1/2 function, which is difficult to evaluate considering the expression of only 4 genes (FLC, MAF4, MAF5 and BSU1).

Thank you for the suggestion. We utilized a threshold of 1.5-fold change and added the suggested analysis to Supplementary Figure 9.

We have also added this paragraph to the results section

"Next, we examined the global effect of nrp1-1 nrp2-2 on transcription. We divided all genes according to expression levels and noted that, generally, only highly-expressed genes were down-regulated by nrp1-1 nrp2-2 ***(Supplementary Fig. 9b)****. This is consistent with previous studies reporting that highly expressed genes have very low H2.A.Z levels while moderately expressed genes have higher H2A.Z levels[14]. We then compared the transcriptomes of arp6-1 and nrp1-1 nrp2-2. Consistent with the phenotype, the overall effect on transcription of arp6-1 was larger than that of nrp1-1 nrp2-2. When comparing both transcriptomes, we observed that the number of genes that were down-regulated in one but up-regulated in the other, (580/317) were marginally higher than the number of genes that were upregulated or downregulated in both (519/272)* ***(Supplementary Fig. 9c)****. These data suggest that ARP6 and NRP proteins may perform a counteracting activity in the cell."*

6- It would be also important to indicate what type of genes are the ones that experience expression change (coding, non-coding, TEs). I am asking because in Supplementary Fig 8, most if not all the genes used to validate the increased levels of H2A.Z in nrp1-1nrp2-2 are TEs.

We are sorry that we did not explain this properly; TEs were excluded from our expression groups analysis since it is hard to define these regions, (promoter, -1, nucleosome, +1 nucleosome, gene body…), in TEs, so there are no TEs in any of the groups.

To clarify this we have now changed the sentence to;

"This analysis showed that the subset of <u>genes annotated as coding</u> with increased H2A.Z occupancy immediately after the TSS (+1 nucleosome), but not other regions, displayed a slight but significant up-regulation in nrp1-1 nrp2-2"

7- It is proposed that the increased levels of H2A.Z leads to overexpression of BSU1, which is consistent with the increased levels of H2A.Z found in nrp1-1nrp2-2 when measured by ChIP-PCR and with the expression of the gene detected in the mutant. According to Fig. 3a, the levels of H2A.Z at BSU1 seem to be high and extended across the gene body in WT, which is consistent with its low expression levels (Fig 1), however, the expression of the gene is not altered in arp6 mutant in which the levels of H2A.Z are reduced, suggesting that the repression of the gene is not affected by a decrease in H2A.Z. The authors indicate that NRP1/2 could play a specific role at low expressed genes in which the increase of H2A.Z occupancy in nrp1-1nrp2-2 takes place immediately after the TSS (+1 nucleosome), but not other regions, is this the case of BSU1? I would recommend to include screenshots of the genomic area of BSU1 showing H2A.Z levels in nrp1-1nrp2-2, arp6 and in the triple mutant.

Yes, *BSU1* is a lowly-expressed gene, (included in Group1), that shows overaccumulation of H2A.Z at the TSS but low H2A.Z levels across the gene body (Figure 3f and Supplementary Figure7).

We appreciate the recommendation. We have now added screenshots showing H2A.Z and NRP1-Myc signal across the mentioned genes in Supplementary Figure 7.

8- In the case of FLC, MAF4 and 5 the effect of nrp1-1nrp2-2 mutation is puzzling.
- In nrp1-1 nrp2-2 mutant, as in arp6-1, FLC expression levels are significantly decreased compared to WT and its expression is not recovered in the triple mutant, indicating that the loss and the gain of H2A.Z levels similarly impact gene expression, which is not consistent with a requirement of H2A.Z for FLC expression. In fact, in nrp1-1 nrp2-2 the levels of H2A.Z seem to be similar to WT and the gene is down regulated. The authors, in an attempt to find a possible explanation for this, propose that the high levels of H3 found at the first intron of FLC in nrp1-1 nrp2-2 double mutant could indicate a higher degree of chromatin compaction that may explain the negative effect of H2A.Z levels in nrp1-1nrp2-2. Is this something that only happens in FLC? Also, as for the case of BSU1, I recommend to include screenshots of the H2A.Z levels at FLC in nrp1-1nrp2-2, arp6 and triple mutant.
- On the other hand, MAF4 and 5 expression levels are recovered in the triple mutant, but what happens with H2A.Z levels, are they recovered? Again, please include screenshots of the H2A.Z levels at MAF4 and 5 in nrp1-1nrp2-2, arp6 and triple mutant.

We agree that the data are complex, and the *FLC* locus is regulated by many epigenetic pathways, and we might be getting some counterintuitive effects there. We have also checked H3 levels at *BSU1* and we have not found any significant difference between *nrp1-1 nrp2-2* and the wild-type. We have added this data in supplementary figure 8.

We have not checked the levels of H2A.Z at *MAF4* and *MAF5* in *arp6-6 nrp1-1 nrp2-2* by ChIP-PCR. But we have done it at *FLC* and *BSU1* and found no differences by ChIP-qPCR. We cannot discard that this is because the differences might be too small to be detected, given that a slight recovery of H2A.Z in the *arp6-1 nrp1-1 nrp2-2* triple mutant background is observable when considering all of the H2A.Z peaks together or *nrp1-1 nrp2-2* dependent peaks (figure 3c and d). Also, we did not find obvious differences when looking at the tracks of individual loci. However, we have to bear in mind that H2A.Z deposition is already impaired in the triple mutant due to the presence of the mutant allele *arp6-1* and a large recovery in H2A.Z abundance is not expected. Nevertheless, genetic data suggest that the slight recovery that might happen could be enough to affect transcription.

We have now added screenshots of *MAF4* and *5* (together with *BSU1* and *FLC*) in Supplementary Figure 7.

9- According to previous data, NRP1 can interact with all canonical histones, and here the authors clearly show an in vivo interaction with H2A.Z. I wonder whether the authors found H2A and/or H2A.Z among the peptides detected in IP-MS experiment using NRP2-Flag and NRP2-Myc. You mentioned that only unique peptides across all replicates are shown, however, if the NRP proteins interact with H2A and H2A.Z, an enrichment of peptides corresponding to these histones should be detected compared to WT Col control. Also, despite the authors found a consistent enrichment of At4g31620 peptides in all IPs, they did not comment anything about this B3 factor. Is there any information about its possible role? Could this interaction fit with NRP function?

Actually, we didn't find any consistent enrichment across all four replicates except for a 3-fold enrichment in H2A.Z, (HTA8, 9 and 11), in the NRP2-9xMyc replicate. Here is a summary of all peptides from Histone2A family across all four replicates

	NRP-3xFlag	NRP2-9xMyc	NRP2-9xMyc	NRP2-9xMyc	Columbia	Columbia	Columbia	Columbia
**HTA1**	0	2	2	3	0	0	2	3
**HTA2**	0	2	2	3	0	0	2	3
**HTA3**	0	0	0	0	0	3	0	0
**HTA4**	0	0	8	14	0	0	9	11
**HTA5**	0	2	0	0	0	5	0	0
**HTA6**	0	0	2	2	0	2	0	2
**HTA7**	0	0	0	0	0	2	0	0
**HTA8**	0	0	8	15	0	0	9	5
**HTA9**	0	0	8	15	0	9	9	5
**HTA10**	0	2	2	3	0	0	2	3
**HTA11**	0	0	8	15	0	0	9	5
**HTA13**	0	0	0	0	0	2	0	0

For Co-IP assays (figure 2a,b and c) we were able to confirm the interaction in both directions, pulling from our tagged proteins and H2A.Z

We are adding the complete list of all the IP experiments and their corresponding controls as supplementary data

With regard to At4g31620, we have not found any information about this gene relevant to the paper.

10- Fig 1f- I would recommend to include the heatmap of H2A.Z peaks in WT together with the one in arp6 and the triple mutant.

Thank you for the suggestion, We are now including heatmaps and plots for all the genotypes in former Figure 3d and f, (now Figure 3c and d)

11- Metagene plot in nrp1-1 nrp2-2 show an increase of H2A.Z along the entire gene, which does not fit with the proposal that the increase in H2A.Z occupancy in nrp1-1nrp2-2 occurs in the region immediately after the TSS (+1 nucleosome), but not other regions (Line 207 to 216). It would be interesting to include the metagene plots of H2A.Z in arp6 and the triple mutant.

We are sorry for the misunderstanding, we might not have explained it correctly, what we meant is that even though an increase in H2A.Z is observed along the entire gene body in metaplots, this increase appears to be more pronounced in lowly expressed genes than in highly expressed genes (Figure 3g). Another observation is that among the subset of genes that shows a highly significant increase in H2A.Z occupancy, (*nrp1-1 nrp2-2* dependent peaks), only those in which the peak is located immediately after the TSS (+1 nucleosome) show a slight upregulation in their expression.

The following sentences are now under two separate paragraphs so they will hopefully not cause confusion.

***nrp1-1 nrp2-2* shows overaccumulation of H2A.Z.**

"The increased occupancy of H2A.Z observed in nrp1-1 nrp2-2 occurred predominantly at lowly-expressed genes ***(Fig. 3e)****, which is consistent with the fact that lowly expressed genes typically show high levels of H2A.Z[14, 34]"*

***arp6-1* and *nrp1-1 nrp2-2* have opposite effects on transcription.**

"…To better understand the effects of NRP proteins on transcription we first focused on the H2A.Z peaks that were dependent on nrp1-1 nrp2-2. This analysis showed that the subset of genes annotated as coding with increased H2A.Z occupancy immediately after the TSS (+1 nucleosome), but not other regions, displayed a slight but significant up-regulation in nrp1-1 nrp2-2 (Supplementary Fig. 9a)*…"*.

We have now also added *arp6-1* and *arp6-1nrp1-1nrp2-2* plots to the H2A.Z and H2A metagene analyses in Figure 3b.

12- It is surprising that the levels of H2A do not change in nrp1-1nrp2-2. If NRP1/2 mitigate the excessive deposition of H2A.Z, thus balancing H2A/H2A.Z levels, a decrease in H2A should be expected in nrp1-1nrp2-2. If NRP1 is mostly bound to heterochromatic regions and H2A.Z increased levels are mostly found at these regions (which I consider the most interesting finding of the manuscript), it might be possible that it acts mitigating the incorporation of H2A.Z at H2A.W domains. It would be interesting to investigate the levels of H2A.W in nrp1-1nrp2-2 or at least whether the regions with increased H2A.Z levels are normally occupied by H2A.W (genome wide localization data of H2A.W are already available).

Thank you, that is an excellent suggestion. We have now plotted H2A.W ChIP signal from Yelagandula *et al*., 2014 over *nrp1-1 nrp2-2* dependent H2A.Z peaks, and it looks like there is not any substantial enrichment, if anything a minor dip can be detected (Supplementary Figure 10).

We have also included this sentence in the manuscript: *"Additionally, H2A.W ChIP signal in the wild-type did not show any enrichment at nrp1-1 nrp2-2 dependent H2A.Z loci"*

13- I recommend to include also screenshots of NRP1 localization at FLC, MAF4, 5 and BSU1.

We have now added screenshots of *BSU1*, *FLC*, *MAF4* and *5* in Supplementary Figure 7

14- The authors indicate that the increased occupancy in nrp1-1nrp2-2 tends to occur in low expressed genes (Group 1, 2 and 3). Which genes are included in this groups? How many genes of these groups are upregulated? Are they TEs? The validated genes are TEs (At1g54760, At4g08090, At5g35935) or close to TEs (At3g42786, At5g33390) genes. Are they group 1 genes? In which group are included FLC, MAF4/5 and BSU1?

As we mentioned above, TEs were excluded from our expression group analysis so there are no annotated TEs in any of the groups.

The genes were divided according to the following FPKM values:

FPKM>30 group5
>=30>15 group4
>=15>6 group3
>=6>1 group2
0~1 group1

The threshold limits used for the division of genes according to their expression have been added to the legend of Supplementary figure 9

We are adding the complete RNA-Seq experiment including, the complete list of genes and their expression in Columbia and *nrp1-1 nrp2-2*, a list of up-regulated genes, a list of down-regulated genes, and all the genes included in every group (Group1 to 5) separately as supplementary data.

The distribution of *BSU1, FLC*, *MAF4* and *5* are as follow:

BSU1 belongs to Group1
FLC belongs to Group3
MAF4 belongs to Group2
MAF5 belongs to Group3

15- In the discussion, the authors contemplate several possibilities to explain the increased levels of H2A.Z in nrp1-1 nrp2-2. The authors favor the hypothesis that NRP1/2 is involved in eviction of H2A.Z, which I consider plausible. In any case, an increase of H2A.Z in the double mutant implies that another H2A is removed. According to the results, it seems that it is not canonical H2A as its levels are not altered in nrp1-1 nrp2-2. In supplementary Figure 6, the authors checked by WB the global levels of H2A.Z in different mutants, it would be interesting to include H2A.X and H2A.W in the analysis. An intriguing possibility that in my opinion deserves to be investigated is that NRP1/2 evict H2A.Z from the regions where H2A.W should be incorporated.

Although this is an excellent suggestion, we unfortunately have not been able to get antibodies against H2A.X due to issues related with the exchanging of biological material between countries. However, we have tested global levels of H2A.W in all four genotypes by western, but found no significant changes (Supplementary Figure 5). We have also added this paragraph to the new version of the manuscript in the results section:

"The overall distribution pattern of both NRP1-Myc and the major gains of H2A.Z in nrp1-1 nrp2-2 double mutants resembled that of the histone variant H2A.W, which is primarily centromeric[37]. In order to explore any possible relationship between NRP proteins and H2A.W, we examined global levels of H2A.W by western blot in Columbia and nrp1-1 nrp2-2 double mutants and did not find any significant changes"

And this sentence to the discussion section:

"Moreover, global levels of H2A.W appear to be unaffected in nrp1-1 nrp2-2 double mutants"

Again, we would like to thank the reviewers for these helpful suggestions and we hope the manuscript is now acceptable for publication.

Reviewers' comments:

Reviewer #1 (Remarks to the Author):

I am happy with the changes made by the author to address my concerns. I feel the changes to address my concerns and those of the other reviewers has substantively improved the manuscript.

Reviewer #2 (Remarks to the Author):

The authors have satisfied my concerns with the previous version of the manuscript.

There are two minor corrections that should be made to the text:

Lines 77-78: "methyl binding protein 9" should be "methyl-CpG-binding domain 9"

Line 98: should be "associate with" rather than "associate to"

Reviewer #3 (Remarks to the Author):

The authors have included new figures to address my previous comments. However, after revising the new information, I am still not convinced with the results obtained with FLC, MAF4/5 and BSU1. While the results showing increased levels of H2A.Z in nrp1/2 around heterochromatic regions and centromeres and NRP1 direct binding at these regions are clear, I found that for FLC, MAF4/5 and BSU1 genome-wide and PCR based results (for both expression and H2A.Z levels) do not support each other. Therefore, even though it might be possible that the loss of NRP1 function has direct effect on transcriptional regulation, the results presented are not solid.
I would suggest to focus the manuscript in those genes that support the main finding. The fact that NRP proteins seem to be involved in maintaining low levels of H2A.Z around centromeres is a novel and exciting finding, thus, I think that the authors should investigate in more detail the genes located at these regions.

Below you will found the comments in which I based my concerns:

1-RNA-seq analysis shows that the expression of BSU1 is considerably affected in nrp1/2 mutant and that FLC expression is two times WT expression in nrp1/2, which is consistent with the qRT-PCR analysis of Fig. 1d and c respectively. Conversely, MAF4/5 expression is not really affected when analyzed by RNA-seq, which is in contrast to qRT-PCR results.

2-Also, in contrast to the qPCR results of H2A.Z levels presented in Fig. 3, Browser views of H2A.Z occupancy at FLC, MAF4/5 and BSU1 do not show significant differences in WT compare to nrp1/2 (Supplementary Fig. 7). Furthermore, I do not appreciate a special enrichment of NRP1 at the regions tested by qPCR compare to other regions of these genes. In any case, it is hard to judge if there is any enrichment at these genes as a screenshot of a negative control for NRP1 binding is missing (for instance At1g76840).

3-According to Fig. 3, when considering all H2A.Z peaks in WT, there is a slight increase of H2A.Z in nrp1/2 compared to WT; however, the authors found that the levels of H2A.Z in nrp1/2 were clearly and significantly affected at 1753 genes. Are those genes coding genes? The authors indicated that the expression analyses were focused on coding genes and that TEs were excluded from the expression groups analysis. However, is this also the case for H2A.Z levels? I am asking because several TEs (like At5g35935 and At4g08090) located around centromeric regions are shown as

representative examples of the genes that gain H2A.Z in Supplementary Fig. 6. The levels of H2A.Z at these genes were low in WT and significantly increased in mutant. These results were confirmed by qPCR (I found an error with At5g07295: the screen shot does not correspond to the gene). In my opinion, the results with these genes are clear, support the main finding of the manuscript and are in agreement with a previous study showing that YFP-NRP1 is able to bind to the chromatin of pericentromeric loci. Furthermore, the other representative genes shown in the figure are also located around centromeres (At5g33390, At1g54760 and At3g42786). Therefore, I do not understand why the authors did not focus on these genes for other analyses. For instance, they did not show whether NRP1 is located at these genes. The authors claim that NRP1 co-localizes with H2A.Z depleted regions, since these genes display low levels of H2A.Z in WT and high in mutant, they are good candidates to be direct targets of NRP1 binding.

4-In Supplementary Fig. 11, the authors show that the levels of CG, CGH and CHH methylation in nrp1/2 mutant are not affected at genes (28,496); however, there is an appreciable increase of CG and CHG methylation at TEs that the authors briefly mention. Do the metagene plots in Supplementary Fig. 11d show the methylation patterns at TEs or at all genes? I am asking because in the legend the number n= 31,189 is more likely to correspond to genes+TEs than to TEs. I guess that the number is a mistake. Please, correct in any case.

If the metagene plots indeed correspond to TEs, did the authors check if the increase in CG and CHG methylation take place at TEs with increased levels of H2A.Z in nrp1/2? Since the increase of H2A.Z in nrp1/2 mutant is observed at pericentrometic regions and these regions are enriched in TEs (in fact several pericentromeric TEs are included as representative examples for gain of H2A.Z), it might be possible the two effects take place at the same regions. It would be interesting to show a metagene plot of H2A.Z levels at the TEs shown in Supplementary Fig. 11. I think that the author should address these questions to evaluate whether the increased CG and CHG methylation and increased H2A.Z levels in nrp1/2 are somehow conected or not.

Reviewer #4 (Remarks to the Author):

There are no methods and references about the IP-MS/Proteomics. Important information was left out, e.g., How many biological and technical replicates were done? What database was used? What criteria were used to select confident identifications? Without these information, no one will be able to reproduce the work. Table 1 just listed unique peptides, but how were those peptides chosen? Do they have 1% FDR? It is essential that the data are deposited at a public repository such as PRIDE.

First, we would like to thank all the reviewers for their suggestions and their useful comments that have greatly contributed to improving our manuscript.

Reviewer #1 (Remarks to the Author):

I am happy with the changes made by the author to address my concerns. I feel the changes to address my concerns and those of the other reviewers has substantively improved the manuscript.

Thank you!

Reviewer #2 (Remarks to the Author):

The authors have satisfied my concerns with the previous version of the manuscript.

There are two minor corrections that should be made to the text:

Lines 77-78: “methyl binding protein 9” should be “methyl-CpG-binding domain 9”

Line 98: should be “associate with” rather than “associate to”

Thank you! We have corrected these mistakes in the new version.

Reviewer #3 (Remarks to the Author):

The authors have included new figures to address my previous comments. However, after revising the new information, I am still not convinced with the results obtained with FLC, MAF4/5 and BSU1. While the results showing increased levels of H2A.Z in nrp1/2 around heterochromatic regions and centromeres and NRP1 direct binding at these regions are clear, I found that for FLC, MAF4/5 and BSU1 genome-wide and PCR based results (for both expression and H2A.Z levels) do not support each other. Therefore, even though it might be possible that the loss of NRP1 function has direct effect on transcriptional regulation, the results presented are not solid.
I would suggest to focus the manuscript in those genes that support the main finding. The fact that NRP proteins seem to be involved in maintaining low levels of H2A.Z around centromeres is a novel and exciting finding, thus, I think that the authors should investigate in more detail the genes located at these regions.

Below you will found the comments in which I based my concerns:

1-RNA-seq analysis shows that the expression of BSU1 is considerably affected in nrp1/2 mutant and that FLC expression is two times WT expression in nrp1/2, which is consistent with the qRT-PCR analysis of Fig. 1d and c respectively. Conversely, MAF4/5 expression is not really affected when analyzed by RNA-seq, which is in contrast to qRT-PCR results.

Yes, in the case of *MAF4* and *MAF5*, there is a discrepancy between genomic data and qRT-PCR. However, we have performed the qRT-PCR in three independent biological replicates (three technical replicates each), and we do find significant differences in the expression of *MAF4* and *MAF5* between the wild-type and *nrp1-1 nrp2-2*. Given that qRT-PCR is a more sensitive technique, and since differences are minor, we favor qRT-PCR data over whole-

genome RNA-Seq data, which is not the best technique to detect slight changes. For instance, the p-values for qRT-PCR are much lower than those for RNA-Seq experiments (table below). However, to be more conservative, we are dropping *MAF4* and *5* from the paper, and focusing on *FLC* and *BSU* in which there are no discrepancies.

RNA-Seq	*MAF4*		*MAF5*		*FLC*		*BSU1*	
	Col	*nrp* double	Col	*nrp* double	Col	*nrp* double	Col	*nrp* double
R1	1.374	0.612	0.877	0.879	1.457	0.403	0.122	1.545
R2	1.017	2.849	1.391	1.631	1.243	0.751	0.066	2.409
R3	1.444	1.738	0.553	0.973	1.33	0.792	0.049	1.918
Avg	1.278	1.733	0.94	1.161	1.343	0.648667	0.079	1.95733333
Ttest		0.607389		0.209893		0.061154		0.01941729
RT-PCR	*MAF4*		*MAF5*		*FLC*		*BSU1*	
	Col	*nrp* double	Col	*nrp* double	Col	*nrp* double	Col	*nrp* double
R1	1	0.556	1	0.357	1	0.037	1	71.523
R2	1	0.462	1	0.09	1	0.104	1	81.03
R3	1	0.401	1	0.205	1	0.095	1	71.304
Avg	1	0.473	1	0.217333	1	0.078667	1	74.619
Ttest		0.007238		0.00962		0.000519		0.00189124

Raw data supporting all the figures, including Figure 2d have now been added as source data file.

2-Also, in contrast to the qPCR results of H2A.Z levels presented in Fig. 3, Browser views of H2A.Z occupancy at FLC, MAF4/5 and BSU1 do not show significant differences in WT compare to nrp1/2 (Supplementary Fig. 7). Furthermore, I do not appreciate a special enrichment of NRP1 at the regions tested by qPCR compare to other regions of these genes. In any case, it is hard to judge if there is any enrichment at these genes as a screenshot of a negative control for NRP1 binding is missing (for instance At1g76840).

In the case of H2A.Z levels, we have not tested occupancy at *MAF4* and *MAF5* directly by qPCR, and as mentioned above we are dropping these genes from the paper. Nevertheless, we examined H2A.Z levels at *FLC*, and *BSU1* by ChIP-qPCR in wild-type and *nrp1-1 nrp2-2* in five biological replicates, three technical replicates each, and the results were a 1.33- and a 1.81-fold increase respectively at an already high H2A.Z region. These small differences are not evident by looking at H2A.Z ChIP-Seq tracks, which we feel can be explained by differences in the sensitivity between ChIP-qPCR and whole-genome ChIP-Seq techniques.

Regarding NRP1 binding to *BSU1*, *FLC*, *MAF4/5*, we do not mean to claim in the manuscript that the regions we tested by ChIP-qPCR were specific nor special; we chose these genomic regions because they were previously described as H2A.Z-rich regions and therefore we hypothesized that NRP1 could bind there. If there is something else we can clarify in the manuscript on this issue, we are happy to do so.

We now have added the tracks corresponding to negative control (=*At1g76840*) to Supplementary Fig. 7.

3-According to Fig. 3, when considering all H2A.Z peaks in WT, there is a slight increase of H2A.Z in nrp1/2 compared to WT; however, the authors found that the levels of H2A.Z in nrp1/2 were clearly and significantly affected at 1753 genes. Are those genes coding genes?

The authors indicated that the expression analyses were focused on coding genes and that TEs were excluded from the expression groups analysis. However, is this also the case for H2A.Z levels? I am asking because several TEs (like At5g35935 and At4g08090) located around centromeric regions are shown as representative examples of the genes that gain H2A.Z in Supplementary Fig. 6. The levels of H2A.Z at these genes were low in WT and significantly increased in mutant. These results were confirmed by qPCR (I found an error with At5g07295: the screen shot does not correspond to the gene). In my opinion, the results with these genes are clear, support the main finding of the manuscript and are in agreement with a previous study showing that YFP-NRP1 is able to bind to the chromatin of pericentromeric loci. Furthermore, the other representative genes shown in the figure are also located around centromeres (At5g33390, At1g54760 and At3g42786). Therefore, I do not understand why the authors did not focus on these genes for other analyses. For instance, they did not show whether NRP1 is located at these genes. The authors claim that NRP1 co-localizes with H2A.Z depleted regions, since these genes display low levels of H2A.Z in WT and high in mutant, they are good candidates to be direct targets of NRP1 binding.

As the reviewer remarks, we have described 1,753 H2A.Z peaks that are significantly higher in *nrp1-1 nrp2-2*. These peaks mainly fall into regions annotated as coding (1,467 out of 1,753). To clarify the matter, we have added the following statement to the manuscript:

"These peaks fall mainly in regions annotated as coding genes (1,467 coding / 286 TEs Vs 1,334 coding / 419 TEs that would be expected from a randomly shuffled control)"

No, we did not exclude the TEs from H2A.Z analysis, all peaks were considered independently of where they fell. We selected the loci we have validated (showed in Supplementary figure 6) according to H2A.Z occupancy fold-change and *p*-value. Since differential occupancy between wild-type and *nrp1-1 nrp2-2* concentrates around centromeres, and this is also the region where TEs are mainly localized, a few TEs happened to be included in the list of validated genes. Nonetheless, this is a small sample of genes we validated (*n*=6), and as we mentioned above, when we consider all of the differentially occupied peaks we do not find any bias towards TEs.

To explain our rationale, we focused on *BSU1* because it was dramatically overexpressed in *nrp1-1 nrp2-2*. We also focused on *FLC*, *MAF4/5*, because they are genes whose expression is known to be sensitive to changes in H2A.Z and are important for plant development.

With regard to NRP1 binding, we feel that is evident, even by looking at whole-genome ChIP-Seq tracks, that NRP1 is enriched at pericentromeric regions. However, as the reviewer remarks, NRP1 binding in the chromosome arms is not so apparent; so, we felt that where individual ChIP-qPCR validation was needed the most was at specific putative targets out in the arms such as *BSU1*, *FLC*, *MAF4/5.*

Thank you for catching the error! Supplementary Figure 6 has been corrected.

4-In Supplementary Fig. 11, the authors show that the levels of CG, CGH and CHH methylation in nrp1/2 mutant are not affected at genes (28,496); however, there is an appreciable increase of CG and CHG methylation at TEs that the authors briefly mention. Do the metagene plots in Supplementary Fig. 11d show the methylation patterns at TEs or at all genes? I am asking because in the legend the number n= 31,189 is more likely to correspond to genes+TEs than to TEs. I guess that the number is a mistake. Please, correct in any case.

If the metagene plots indeed correspond to TEs, did the authors check if the increase in CG

and CHG methylation take place at TEs with increased levels of H2A.Z in nrp1/2? Since the increase of H2A.Z in nrp1/2 mutant is observed at pericentrometic regions and these regions are enriched in TEs (in fact several pericentromeric TEs are included as representative examples for gain of H2A.Z), it might be possible the two effects take place at the same regions. It would be interesting to show a metagene plot of H2A.Z levels at the TEs shown in Supplementary Fig. 11. I think that the author should address these questions to evaluate whether the increased CG and CHG methylation and increased H2A.Z levels in nrp1/2 are somehow conected or not.

Thank you for asking. That was actually not a mistake, the TAIR10 release includes 31,189 TEs.

Thank you for this suggestion. As requested we have taken the same group of TEs and indeed we do see an increase in H2AZ enrichment at these sites in nrp1/2 mutants. This has been added as Supplementary Figure 11e.

we also have added this sentence to the main text:

*"Moreover, H2A.Z abundance at TEs appear to be slightly higher in nrp1-1 nrp2-2 than in the wild-type **(Supplementary Fig. 11e).**"*

Reviewer #4 (Remarks to the Author):

There are no methods and references about the IP-MS/Proteomics. Important information was left out, e.g., How many biological and technical replicates were done? What database was used? What criteria were used to select confident identifications? Without these information, no one will be able to reproduce the work. Table 1 just listed unique peptides, but how were those peptides chosen? Do they have 1% FDR? It is essential that the data are deposited at a public repository such as PRIDE.

We have done four biological replicates (one using NRP2-3xFlag, and three more using NRP2-9x-Myc). Please see table 1

We apologize for the lack of methods. We are using the same methods as in many of our recent papers. We have also added this paragraph to the methods section:

"TCA precipitates were digested by the sequential addition of lys-C and trypsin proteases. The digested peptides were fractionated online using reversed-phase chromatography and eluted directly into a Fusion Lumos mass spectrometer (Thermofisher, USA) where MS/MS spectra were collected. Database searching was performed with the SEQUEST algorithm against a protein database downloaded from The Arabidopsis Information Resource. Peptide identification and protein mapping were performed using the DTASelect2 algorithm. Peptide spectrum matches were filtered using false-positive rate of less than 5% as estimated by a decoy database strategy and confident protein identifications required at least two unique peptides per protein. Normalized spectral abundance factor (NSAF) values were calculated by normalizing total spectral counts by protein length and the number of confidently identified spectra in a given analysis. The proteomics data are deposited in the MassIVE data repository (https://massive.ucsd.edu) under the identifier MSV000084979"

The proteomics data are deposited in the MassIVE data repository (https://massive.ucsd.edu)

Username = MSV000084979_reviewer

Password = Ausin

Again, we are grateful to all reviewers for their comments and hope the manuscript is now acceptable.

Reviewer #3 (Remarks to the Author):

The authors have satisfied my concerns with the previous version of the manuscript. However, there are some minor corrections and I have some suggestions:

1. Remove Simbol ! from Fig. 1 legend.

2. Line 126-… in Columbia, nrp1-1, nrp2-2, and the nrp1-1 nrp2-2 double mutant. In contrast with previous transcriptomic analyses using nrp1-1 nrp2-128 we found that transcript levels of BRI1 SUPPRESSOR 1 (BSU1) were dramatically increased in nrp1-1 nrp2-2 double mutants relative to wild-type plants…

A would suggest to change this paragraph to:
… in Columbia, nrp1-1, nrp2-2, and the nrp1-1 nrp2-2 double mutant. Among the misregulated genes, we found that BRI1 SUPPRESSOR 1 (BSU1) transcript levels were dramatically increased in nrp1-1 nrp2-2 double mutants relative to wild-type plants, which was in contrast with previous transcriptomic analyses using nrp1-1 nrp2-1(28).

3. Line 148: Unexpectedly, of BSU1 were….
"Expression levels" or "transcript levels" is missing

4. "….BSU1 were restored to nearly wild-type levels in the arp6-1 nrp1-1 nrp2-2 triple mutant compared to the nrp1-1 nrp2-2 double mutant, and the arp6-1 single mutant."
BSU1 levels are similar in WT and arp6-1 mutant, therefore I think that you should remove "and the arp6-1 single mutant".

5. Please, include in Fig.3c and 3b n = 1,753 and n = 21,024, respectively

6. Line 225:… fact that lowly expressed genes typically show high levels of H2A.Z14, 34
The dot at the end of the sentence is missing.

7. The color code indicating low-high expression levels of the gene groups in Fig.3e is not observed in the figure. Same for Suppl. Fig.9b.

8. Supplementary Fig. 7- Please, show data set range of H2A.Z ChIP and NRP1 ChIP browser view at BSU1.

Reviewer #4 (Remarks to the Author):

There are still errors and improvements to be made related to the mass spectrometry analyses. 1) The added method is too brief. As stated in the authors' response, their previous paper with detailed method should be cited here. 2) Table 1 numbers are likely to be just PSMs, they are not unique peptides. There are too many to be true for NPRs. They are also not NSAFs as described in the method. Please see this paper about NSAFs (https://www.ncbi.nlm.nih.gov/pmc/articles/PMC3599300/). The result in this table was used to support heteromer formation because of nearly 1:1 ratio between NPR1 and NPR2. But heteromer can be formed when the components are not in 1:1 ratio.

In addition, the authors need to proof-read carefully and avoid typos and grammatical errors (for example, indebted to, not indebted with).

REVIEWER COMMENTS

We would like to thank the reviewers for their suggestions and comments.

Reviewer #3 (Remarks to the Author):

The authors have satisfied my concerns with the previous version of the manuscript. However, there are some minor corrections and I have some suggestions:

1. Remove Simbol ! from Fig. 1 legend.

Thanks for noticing the typo, we have removed the exclamation symbol from Figure 1.

2. Line 126-... in Columbia, nrp1-1, nrp2-2, and the nrp1-1 nrp2-2 double mutant. In contrast with previous transcriptomic analyses using nrp1-1 nrp2-128 we found that transcript levels of BRI1 SUPPRESSOR 1 (BSU1) were dramatically increased in nrp1-1 nrp2-2 double mutants relative to wild-type plants...

A would suggest to change this paragraph to:
... in Columbia, nrp1-1, nrp2-2, and the nrp1-1 nrp2-2 double mutant. Among the misregulated genes, we found that BRI1 SUPPRESSOR 1 (BSU1) transcript levels were dramatically increased in nrp1-1 nrp2-2 double mutants relative to wild-type plants, which was in contrast with previous transcriptomic analyses using nrp1-1 nrp2-1(28).

Thank you for the suggestion, we have now changed that sentence as suggested.

3. Line 148: Unexpectedly, of BSU1 were....
"Expression levels" or "transcript levels" is missing

Thanks, we have corrected this mistake

4. "....BSU1 were restored to nearly wild-type levels in the arp6-1 nrp1-1 nrp2-2 triple mutant compared to the nrp1-1 nrp2-2 double mutant, and the arp6-1

single mutant."
BSU1 levels are similar in WT and arp6-1 mutant, therefore I think that you should remove "and the arp6-1 single mutant".

Thanks, we have removed these words in the new version

5. Please, include in Fig.3c and 3b n = 1,753 and n = 21,024, respectively

Thanks, we have added these numbers to the new version

6. Line 225:... fact that lowly expressed genes typically show high levels of H2A.Z14, 34
The dot at the end of the sentence is missing.

Thank you, we have now corrected that mistake

7. The color code indicating low-high expression levels of the gene groups in Fig.3e is not observed in the figure. Same for Suppl. Fig.9b.

Thanks, we have now removed the uninformative color code triangles

8. Supplementary Fig. 7- Please, show data set range of H2A.Z ChIP and NRP1 ChIP browser view at BSU1.

Thank you, we have added the set range at *BSU1* to the new version of Supplementary figure 7

Reviewer #4 (Remarks to the Author):

There are still errors and improvements to be made related to the mass spectrometry analyses.

1) The added method is too brief. As stated in the authors' response, their previous paper with detailed method should be cited here.

We are sorry about the lack of references; the following references have been added to the methods to illustrate the precise way Mass-Spec analyses were performed:

50. Heinzelman P, Powers DN, Wohlschlegel JA, John V. Shotgun Proteomic Profiling of Bloodborne Nanoscale Extracellular Vesicles. Methods Mol Biol 1897, 403-416 (2019).

51. Levy MJ, Washburn MP, Florens L. Probing the Sensitivity of the Orbitrap Lumos Mass Spectrometer Using a Standard Reference Protein in a Complex Background. J Proteome Res 17, 3586-3592 (2018).

52. Xu T, et al. ProLuCID: An improved SEQUEST-like algorithm with enhanced sensitivity and specificity. J Proteomics 129, 16-24 (2015).

53. Cociorva D, D LT, Yates JR. Validation of tandem mass spectrometry database search results using DTASelect. Curr Protoc Bioinformatics Chapter 13, Unit 13 14 (2007).

54. Elias JE, Gygi SP. Target-decoy search strategy for increased confidence in large-scale protein identifications by mass spectrometry. Nat Methods 4, 207-214 (2007).

55. Li H, et al. The Sequence Alignment/Map format and SAMtools. Bioinformatics 25, 2078-2079 (2009).

2) Table 1 numbers are likely to be just PSMs, they are not unique peptides. There are too many to be true for NPRs. They are also not NSAFs as described in the method. Please see this paper about NSAFs

We are very sorry about this mistake, we have now changed the footnote of Table 1 to:

"Total number of identified peptide spectra matched for each protein"

(https://www.ncbi.nlm.nih.gov/pmc/articles/PMC3599300/). The result in this table was used to support heteromer formation because of nearly 1:1 ratio between NPR1 and NPR2. But heteromer can be formed when the components are not in 1:1 ratio.

Thank you for your clarification, we have changed that sentence to:

"which is consistent with these two proteins forming heteromers in vivo[34]*"*

In addition, the authors need to proof-read carefully and avoid typos and grammatical errors (for example, indebted to, not indebted with).

Thanks, we have corrected that mistake and quite a few others

Again, we appreciate the comments from all reviewers and hope the manuscript is now acceptable.

REVIEWERS' COMMENTS:

Reviewer #4 (Remarks to the Author):

The authors have addressed my concerns.
